# Identification of SARS-CoV-2-binding lectins on a commercial lectin array

**DOI:** 10.1038/s41598-025-01903-5

**Published:** 2025-07-01

**Authors:** Neetu, Shimona Ahlawat, Rathina Delipan, Rajesh P. Ringe, Alka Rao, T. N. C. Ramya

**Affiliations:** 1 CSIR- Institute of Microbial Technology, Sector 39-A, Chandigarh, 160036 India; 2 Academy of Scientific & Innovative Research (AcSIR), Ghaziabad, Uttar Pradesh 201002 India; 3 Present Address: Food Safety and Standards Authority of India (FSSAI), New Delhi, 110002 India

**Keywords:** SARS-CoV-2, Lectins, Spike, Glycosylation, Lectin array, Biochemistry, Biological techniques, Biotechnology, Microbiology, Molecular biology

## Abstract

The Spike glycoprotein of SARS-CoV-2 is the major target for vaccines and therapeutics. Spike glycosylation is critical for ACE2 binding and subsequent viral fusion and entry. Here, we studied lectins for their ability to bind to SARS-CoV-2 Spike glycoprotein and SARS-CoV-2 virions by employing an array of 95 lectins, for 68 of which we predicted glycan-binding specificities using publically available glycan array data and MotifFinder software. We identified lectins with diverse glycan binding specificities that bound with high intensities to recombinant Spike and cultured SARS-CoV-2 virus – AAL, ABL, ACL, AMA, ASA, BANLEC, BC2L-A, RCA 120, CALSEPA, GAL3, GS-II, PALa, CA, HHA, PHA-L, PA-IIL, MNA-M, STL, LSL-N, GRFT, PSA, RS-FUC, PHA-E, CPA, LENTIL, RCA 60, GNA, ORYSATA, LcH A, PHA-P, PTL-2, MAA, Con A, TL, NPA, and SBA. Analyzing the glycan-binding specificities of these lectins, we predict that the Spike glycoprotein is modified with high mannose/hybrid N-glycans with terminal mannose residues, α1-6 core fucosylated N-glycans with terminal GlcNAc residues, and complex glycans with Lewis A, Lewis B, Lewis X, Lewis Y, and Blood group H structures on type-1 or type-2 extension sequences. The SARS-CoV-2-specific lectins identified in our study may be assessed for their antiviral potential in future studies.

## Introduction

The Spike glycoprotein of the SARS-CoV-2 virus, the infectious agent of the coronavirus disease 2019 (COVID-19) pandemic, has emerged as a pivotal focal point in the scientific understanding of the virus and its pathogenicity. This trimeric glycoprotein, abundantly displayed on the viral envelope, plays a critical role in the initial stages of infection by facilitating viral entry into host cells by binding to the ACE2 receptor^1–4^.

Structurally, the monomer Spike protein comprises two distinct subunits, the S1 subunit (14–685 aa) and the S2 subunit (686–1273 aa), linked with a furin cleavage site and a transmembrane protein serine protease 2 (TMPRSS2) site^5–7^. The S1 subunit, housing the N-terminal domain and a receptor-binding domain (RBD), is responsible for recognizing and binding to the host cell receptor ACE2, a crucial step in viral attachment. The S2 subunit, on the other hand, contains a fusion peptide, central helix, connected domain, heptad repeat domain, transmembrane domain, and a cytoplasmic tail, which orchestrates membrane fusion, enabling the virus to enter the host cell^2,7^. The trimeric Spike protein is heavily glycosylated and harbors 66 N-linked glycosylation sites (22 sites per monomer) and variable O-glycan sites^8,9^.

Besides providing structural stability, glycosylation provides viruses a shield to escape the host’s immune response and maintain infection^9–11^, Spike glycosylation of SARS-CoV-2 accounts for 17% of molecular weight and 40% of the protein surface^12^. Around 79% of glycans are complex-type glycans, which are bi-antennary, tri-antennary, or tetra-antennary with or without bisecting GalNAc, and around 21% of N-glycosylation sites have high mannose or hybrid content in the S1 subunit of Spike protein isolated from SARS-CoV-2 infected Calu3 cells^13^. N-glycosylation sites are conserved in all variants of concern except for the addition of the N188 site in the Gamma variant and the removal of the N17 site in the Delta variant^14^, reflecting the necessity of glycosylation for viral binding, infectivity, and transmission. The glycosylation sites, N331 and N343, are present in the RBD region and hence crucial for viral infectivity, whereas the N165 and N234 glycosylation sites present adjacent to the RBD region are responsible for the conformational change of RBD from the closed to the open conformation, which facilitates the receptor engagement to the ACE2^15,16^. Thus, the extensive glycosylation of Spike protein influences its structural stability, immune recognition, and receptor binding affinity, which are fundamental to understanding SARS-CoV-2 infectivity, immune evasion, and the development of therapeutic interventions^17–19^.

While the glycan shield presents a formidable challenge in vaccine and therapeutic development by concealing critical epitopes, it also offers opportunities^20,21^. By dissecting the composition and function of the glycan shield, researchers can design strategies to target conserved regions that are less shielded. Albeit researchers have employed mass spectrometry to extensively probe the monosaccharide composition of the glycan shield on the SARS-CoV-2 Spike glycoprotein in domain constructs, trimeric protein, and intact virions, these studies are missing one critical data component, i.e., the glycosidic linkages present in these glycans.

**Fig. 1:**
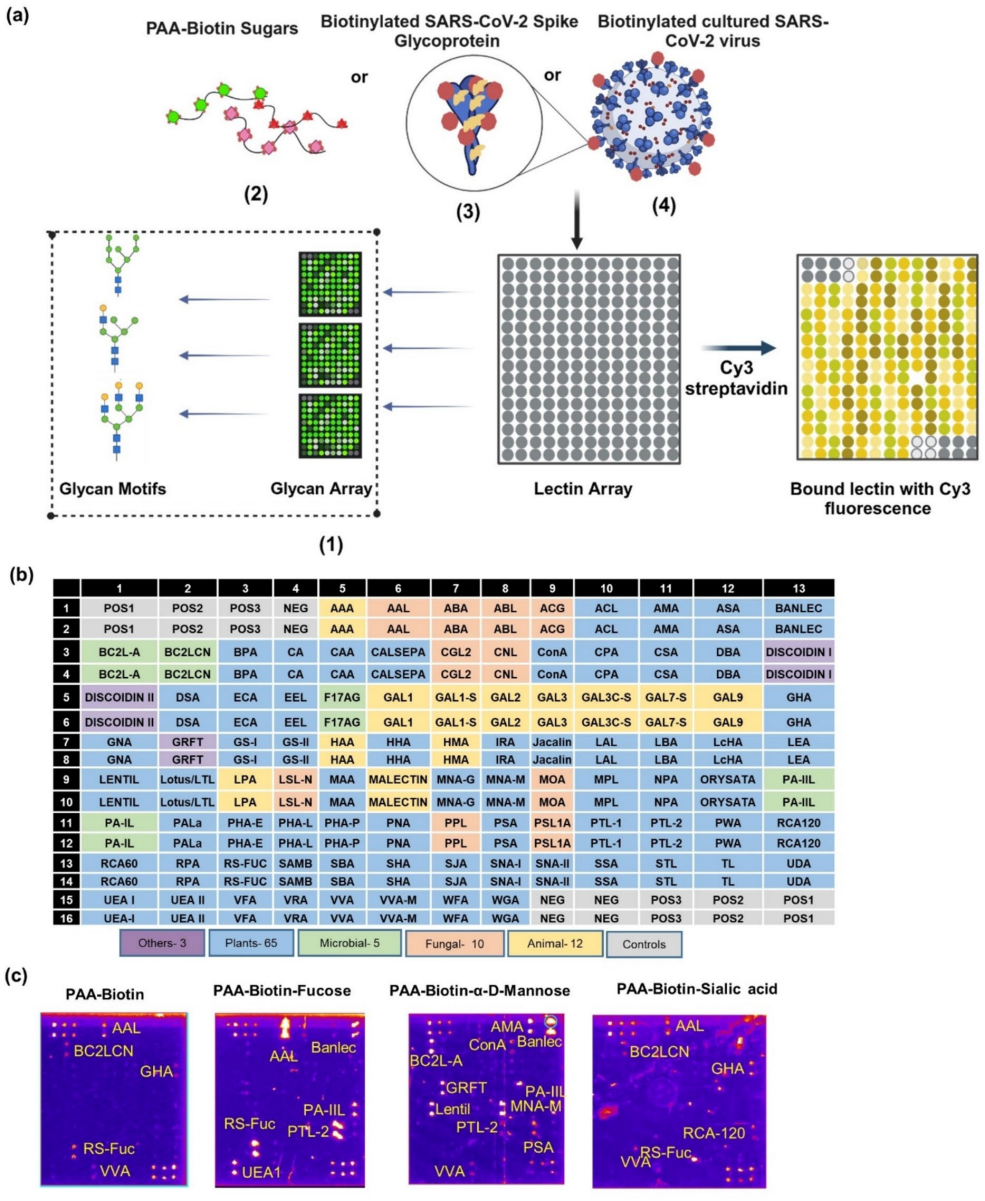
Lectin array: strategy, overview, and validation. **(a)** The glycan binding profile of 68 of the 95 lectins on the microarray was analyzed using the publically available glycan array data and the MotifFinder tool (1). Then, PAA-Biotin sugars (2) or NHS-biotinylated SARS-CoV-2 Spike glycoprotein (3) or NHS-biotinylated SARS-CoV-2 cultured virus (4) was incubated on the lectin array, followed by the addition of Cy3 equivalent dye-streptavidin, and the binding to lectins assessed by measurement of Cy3 fluorescence. Created with BioRender.com **(b)** The lectin array slide (Ray Biotech) consists of 95 lectins printed in duplicates, 12 positive controls, and 6 negative controls. These lectins are from various sources – plants (blue), microbial (green), fungal (orange), animal (yellow), slime moulds and red algae (purple). Controls are colored grey. **(c)** A pseudo-coloured image showing the binding of PAA-Biotin, PAA-Biotin-α-L-fucose, PAA-Biotin-α-D-Mannose, and PAA-Biotin-α-Neu5Ac to the lectins on the lectin microarray. The image was generated using the Protein Array Analyzer macro plugin in Image J.

Lectins, which are non-immune and non-enzymatic glycan-binding proteins, bind to glycan motifs with specific linkages and hence present a viable option to dissect the glycosidic linkages present within any glycoconjugate. By binding to specific glycans on the surface of viruses, lectins can also block virus attachment or interfere with virus entry at an early stage of infection; their ability to target essential steps in the virus life cycle makes them promising candidates for prophylactic and therapeutic interventions against viral infections^22,23^. Several lectins, such as Griffithsin^24^, banana lectin^25^, Wheat Germ Agglutinin (WGA)^26^, *Pholiota squarrosa* lectin (PhoSL)^27^, Lectin FRIL^28^, UDA^29^, and lentil lectin^30^ have been shown to have anti-viral activity against SARS-CoV-2. Here, we have used a commercial lectin array to screen 95 lectins from different sources of origin and with different glycan binding profiles for their potency to bind to the glycans on SARS-CoV-2 Spike glycoprotein and SARS-CoV-2 virions (Fig. 1a). We chose the Wuhan SARS-CoV-2 strain as the representative model for this study because glycans are differentially expressed on variants of concern even though glycosites are conserved^14,31^, and several studies have employed mass spectrometry to study glycosylation in the Wuhan SARS-CoV-2 strain^9,32–36^ with consensus between the glycan profiles obtained in recombinant stabilized Spike (2P) glycoprotein preparations from different laboratories and viral derived Spike protein^13,33,34^, and between recombinant stabilized Spike (2P) and recombinant stabilized Spike (6P/HexaPro)^37^. We used recombinant Wuhan SARS-CoV-2 HexaPro Spike Glycoprotein Ectodomain expressed and purified from Expi293 F™ cultures and SARS-CoV-2 virions propagated from Vero E6 cells for this study. We identify several lectins that may be explored for their diagnostic and therapeutic potential in future studies and use the predicted binding profiles of the lectins to shed some insights into the glycosidic linkages present within the glycan shield of the Spike glycoprotein.

## Results

### Glycan binding profiles of lectins on the lectin array

We used a commercial lectin array comprising 95 lectins for this study (Fig. 1b). The 95 different lectins are spotted in duplicates, and the array also contains six pairs of positive control spots (indicative of binding to streptavidin) and three pairs of negative control spots (that do not show any signal upon incubation with streptavidin). The 95 lectins include 5 microbial lectins, 12 animal lectins, 65 plant lectins, 10 fungal lectins, 2 slime mold lectins, and 1 red algal lectin (Fig. 1b), and the broad specificities of these lectins are available in published literature and indicated by the manufacturer.

For a preliminary validation of the lectins on the array and the lectin array assay protocol adopted, we used biotinylated polyacrylamide probes with or without derivatization with the monosaccharides, mannose, fucose and sialic acid (PAA-Biotin, PAA-Biotin-α-D-Mannose, PAA-Biotin-α-L-Fucose, and PAA-Biotin-α-Neu5Ac), and saw that visible levels of non-specific binding to PAA-Biotin was observed for the lectins, AAL, VVA, RS-FUC, GHA and BC2LCN (Fig. 1c). High binding intensities exceeding that observed with PAA-Biotin (fold change > 3 for RS-FUC and AAL; fold change > 30 for the other lectins) and exceeding that of the positive control spots (relative percent binding intensity > 100%) were observed for the “L-fucose binding lectins”, AAL, PTL-2, RS-FUC, UEA-I, and PA-IIL, and for PWA, as well as for the “mannose-binding lectin” BANLEC when incubated with PAA-Biotin-α-L-fucose (Fig. 1c and Supplementary Data 1). The “L-fucose binding lectins”, LAL and AAA had lower binding intensities (relative percent binding intensity > 5%) that nevertheless exceeded that observed with PAA-Biotin (fold change > 3); other “L-fucose binding lectins”, BC2LCN, Lotus, and LBA had low binding intensities that did not significantly exceed that observed with PAA-Biotin (Supplementary Data 1 of Fig. 1c).

Similarly, high binding intensities exceeding that observed with PAA-Biotin (fold change > 30) and exceeding that of the positive control spots (relative percent binding intensity > 100%) were observed for the “mannose-binding lectins”, BANLEC, MNA-M, AMA, BC2L-A, Lentil, and GRFT, and for PTL-2, PA-IIL, and LAL when incubated with PAA-Biotin-α-D-Mannose (Fig. 1c and Supplementary Data 1). The “mannose-binding lectins”, CGL2, PSA, HHA, NPA, ASA, GS-I, ORYSATA had lower binding intensities (relative percent binding intensity > 5%) that nevertheless exceeded that observed with PAA-Biotin (fold change > 3); other “mannose-binding lectins”, VVA-M, VFA, PHA-L, PHA-E, PHA-P, PALa, CPA, LcHA had low binding intensities that did not significantly exceed that observed with PAA-Biotin (Supplementary Data 1 of Fig. 1c).

High binding intensities exceeding that observed with PAA-Biotin (fold change > 30) and exceeding that of the positive control spots (relative percent binding intensity > 100%) were observed for the “sialic acid binding lectin”, SNA-I, and for DBA and Lentil lectin, when incubated with PAA-Biotin-α-Neu5Ac (Fig. 1c and Supplementary Data 1). Other “sialic acid-binding lectins,” SAMB, LPA, PSL1 A, and ACG, did not bind to PAA-Biotin-α-Neu5Ac (Fig. 1c and Supplementary Data 1).

The non-typical binding and the absence of binding observed for some of these lectins might be due to the multivalent presentation of the monosaccharide and the low affinity for the monosaccharide presented on the PAA glycoconjugates, respectively.

**Fig. 2:**
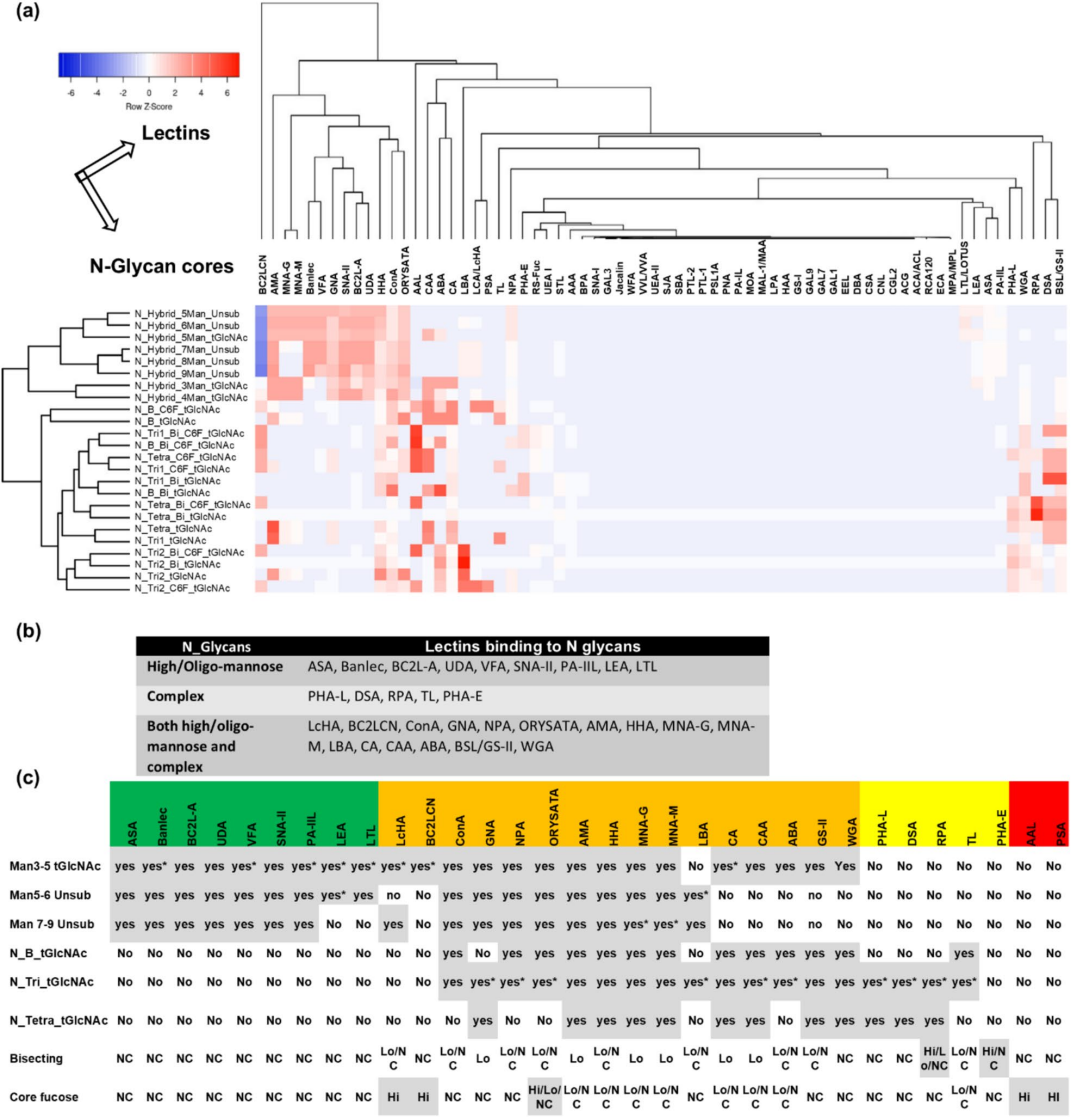
Predicted binding of lectins to N-core glycans. **(a)** A heat map showing the predicted binding propensities of 68 lectins (portrayed on the columns) to different N-cores (portrayed on the rows). The Z-score is a measure of the number of standard deviations between an individual data value and the mean, and is given by the formula, Z = (X-µ)/σ, where X is the individual data value, µ is the population mean, and σ is the population standard deviation. **(b)** Lectins categorized as per their propensities to bind to high mannose glycans and complex glycans. **(c)** Detailed categorization of lectins based on their predicted binding propensities to different N-cores with and without bisecting, with and without branching, and with and without core fucosylation. NC: No change, HI: Increased binding, Lo: Decreased binding, Yes*: Binding to some but not all specified cores under that category.

**Fig. 3:**
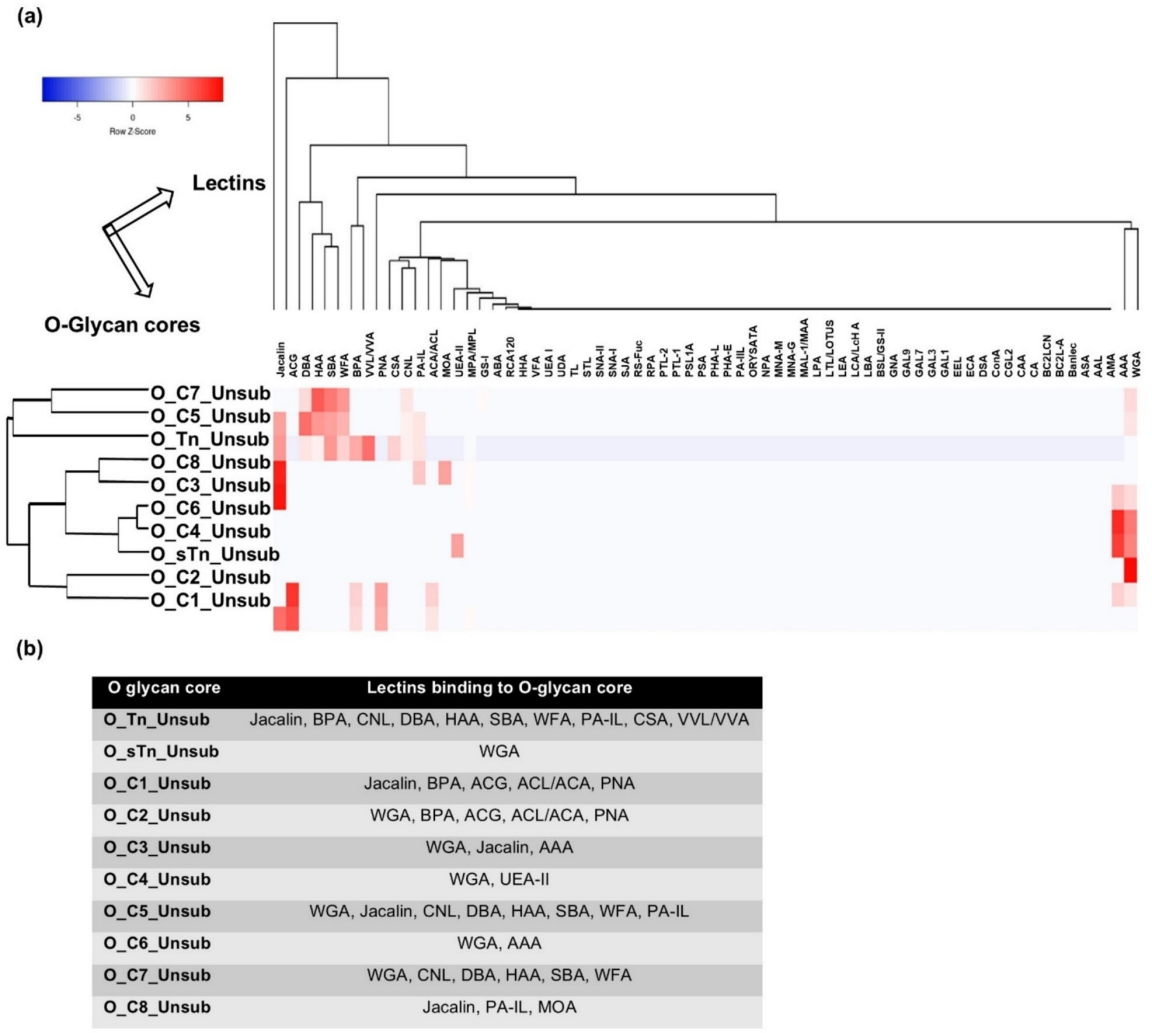
Predicted binding of lectins to O-core glycans. **(a)** A heat map showing the predicted binding propensities of 68 lectins (portrayed on the columns) to different O-cores (portrayed on the rows). The Z-score is a measure of the number of standard deviations between an individual data value and the mean, and is given by the formula, Z = (X-µ)/σ, where X is the individual data value, µ is the population mean, and σ is the population standard deviation. **(b)** Lectins categorized as per their propensities to bind to different O-cores.

We sought to more thoroughly predict the glycan binding propensities of the lectins on this array so that we could have reasonable expectations as to the binding of recombinant Spike glycoprotein or cultured SARS-CoV-2 virus to lectins on this array. We did this by employing glycan array data. Publically available glycan array datasets existed for 68 of these 95 lectins, and the glycan binding specificities of these lectins were also available on Carbogrove^38^. To have a unified analysis of all these lectins, we employed the MotifFinder tool using all available glycan array data for each of the lectins and the same settings of MotifFinder, and generated a lectin model for each of these 68 lectins (Supplementary Data 2). Following this, we generated an in-silico list of core and extension sequences of N- and O-glycans expected to be found on mammalian cells and used the lectin models obtained in the previous step to generate binding propensities, i.e., predicted binding intensities of each of these 68 lectins to these virtual N-glycans and O-glycans (Figs. 2–3, and Supplementary Data 1).

Out of the 68 lectins for which we predicted glycan binding propensities, 17 lectins were predicted to bind to the O-glycan core, 32 lectins were predicted to bind to the N-glycan core, and 18 lectins were predicted to bind to extension sequences (N-glycan extensions and O-glycan extensions) (Figures S1a, S1b, Table S1, Supplementary Data 1). MPA did not show significant predicted binding to any of the generated glycans (Figures S1a, S1b, Table S1, Supplementary Data 1).

### Lectins predicted to bind to N-core and N-extension sequences

The generated heat map of the binding propensities for N-glycan core sequences (portrayed on the rows) showed that some lectins (portrayed on the columns) such as MNA-M, MNA-G, BANLEC, AAL, CAA, VFA, DSA, AMA, ABA, and UDA exhibit high predicted binding intensities towards N-glycans whereas other lectins like Jacalin, SBA, SJA, WFA, EEL, GAL7, and MOA have lower binding propensities for N-glycans (Fig. 2a and Supplementary Data 1). Lectins predicted to bind to the N-glycan core were grouped in three clusters—lectins predicted to bind to the high mannose-containing glycans, lectins predicted to bind to complex type N-glycans, and lectins predicted to bind to both mannose-containing glycans as well as complex type N-glycans. Using a binding propensity cutoff of 0.1, we could classify the lectins into three discrete groups (Fig. 2b). ASA, BANLEC, BC2L-A, UDA, VFA, SNA-II, PA-IIL, LEA, and LTL were predicted to bind to only high/oligo-mannose N-glycan cores (Man 3–5 terminal GlcNAc (Man 3–5 tGlcNAc), Man 5–6 unsubstituted, and Man 7–9 unsubstituted binding lectins) (Figs. 2b, 2c). Lectins like PHA-L, DSA, RPA, TL, and PHA-E were predicted to bind to only complex-type N-glycan cores (bi-antennary N-glycans with tGlcNAc, tri-antennary N-glycans with tGlcNAc, and tetra antennary N-glycans with tGlcNAc) (Figs. 2b, 2c). The lectins, LcHA, BC2LCN, ConA, GNA, NPA, ORYSATA, AMA, HHA, MNA-G, MNA-M, LBA, CA, CAA, ABA, BSL/GS-II, and WGA were predicted to bind to both high/oligo-mannose and complex-type N-glycan cores (Figs. 2b, 2c). Using the binding propensities, we also determined the effect of branching and core-fucosylation. PHA-E was predicted to bind only to complex-type N-glycans with or without bisecting GlcNAc branch, besides being predicted to bind to extension sequences (with higher binding propensities) (Figs. 2c, S1a, S1b, Table S1, Supplementary Data 1). RPA displayed increased binding propensity for some N-glycan cores with a bisecting GlcNAc branch. LcHA, ConA, ORYSATA, NPA, HHA, LBA, ABA, GS-II, and TL showed decreased binding propensity for N-glycan cores with a bisecting GlcNAc branch (Fig. 2c). PSA and BC2LCN were predicted to bind only to core-fucosylated complex-type N-glycan cores and were not predicted to bind to any extension sequences (Figs. 2c, S1a, S1b, Table S1, Supplementary Data 1). AAL and LcHA displayed increased binding propensities for some core-fucosylated N-glycans. Most lectins, with the exception of BC2LCN, DSA, GNA, LcHA, LEA, PHA-L, PSA, RPA, SNA-II, and VFA, were also predicted to bind to N-extension sequences (Figure S1a, Table S1 and Supplementary Data 1).

### Lectins predicted to bind to O-core and O-extension sequences

The generated heat map of the binding propensities for O-glycan core sequences identified Jacalin, SBA, ACG, HAA, WFA, DBA, BPA, PNA, PA-IL, CNL, ACL, AAA, and WGA as lectins with high binding propensities for O-glycan core sequences (Fig. 3a and Supplementary Data 1). Many of these lectins were predicted to bind more than one type of O-core. The exceptions were CSA and VVA, which were predicted to bind only the Tn antigen; UEA-II, predicted to only bind the O-glycan core-4; and MOA, predicted to bind only the O-glycan core-8. Conversely, all cores were predicted to be bound by more than one lectin, with the exception of sialylated Tn antigen, which was predicted to be bound only by WGA. The predicted binding of lectins to the various types of O-glycan cores is shown in Fig. 3b. With the exception of CSA and VVA, all these lectins were also predicted to bind to O-extension sequences (Figure S1b, Table S1 and Supplementary Data 1).

### Lectin binding profiles

Based on the MotifFinder output of the publically available glycan array data, we could compile the predicted fine-tuned carbohydrate specificity of most lectins, barring a few exceptions (Table S2 and Supplementary Data 1). Glycan motifs were not predicted with confidence for a few lectins such as PNA and HAA, and motifs radically different from the carbohydrate specificity as per the Ray Biotech lectin array manual and/or accepted knowledge were predicted for a few lectins. For instance, for AAA and LTL, the MotifFinder output indicated terminal β-GlcNAc non-N-glycan as the preferred motif rather than fucosylated motifs, probably arising from the inclusion of synthetic glycans comprising GlcNAc repeats in the CFG glycan arrays for which the binding intensity was very high (Table S2). Other lectins for which we obtained atypical results were MNA-G (the glycan motif was high mannosyl core instead of galactosylated glycans), LPA (the glycan motif was terminal α-GlcNAc and type 2 TriLacNAc/I antigen instead of sialylated glycans), and UDA (the glycan motif was terminal mannose containing glycans that also had core GlcNAc instead of just GlcNAc containing glycans) (Table S2).

### Binding of lectins to Spike glycoprotein of SARS-CoV-2

**Fig. 4:**
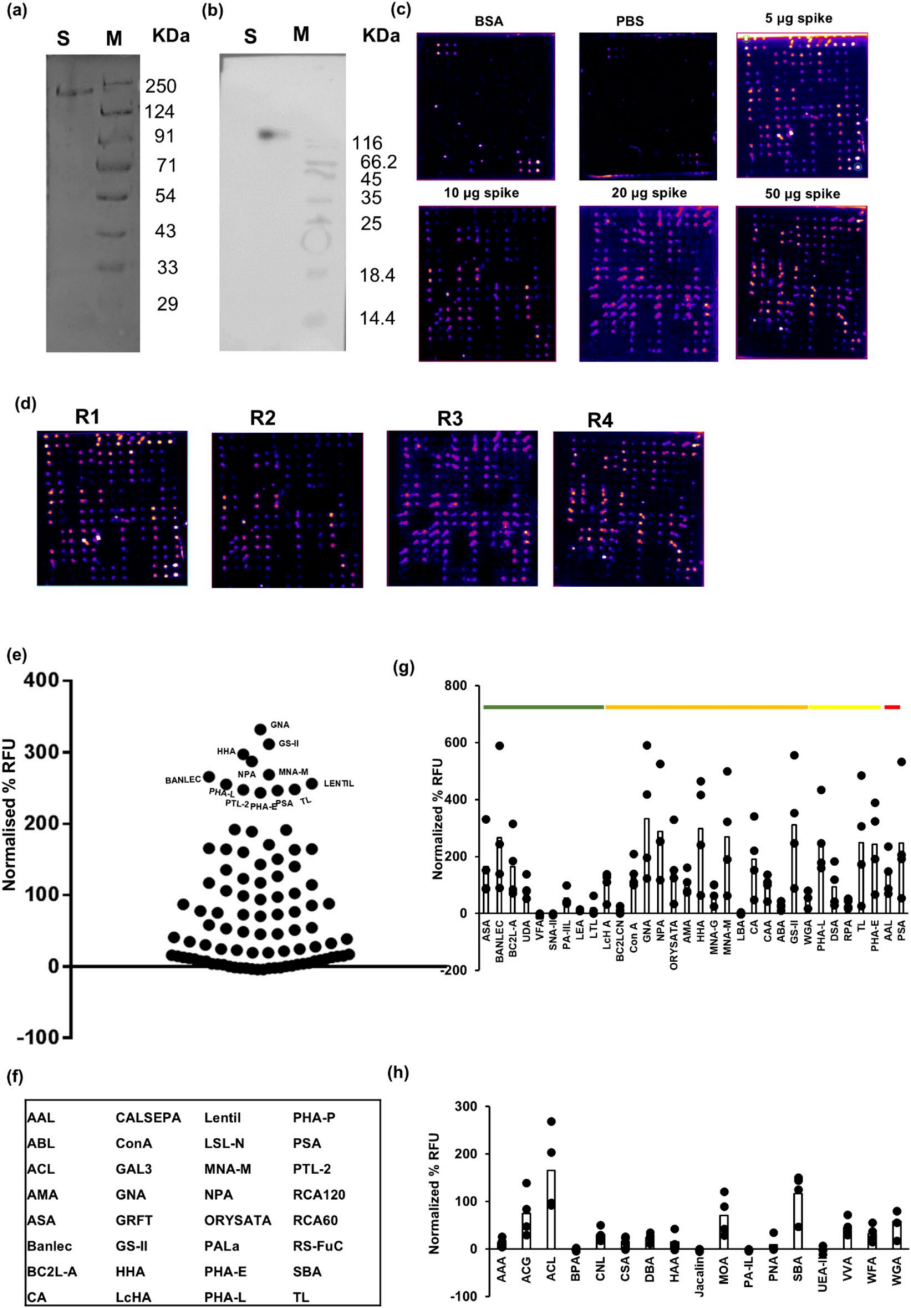
Binding of lectins to SARS-CoV-2 Spike glycoprotein. **(a)** SDS-PAGE of Spike glycoprotein (~ 140 KDa) expressed and purified from Expi293 F™ cells. **(b)** Western blot of purified Spike glycoprotein expressed and purified from Expi293 F™ cells. The blot was developed using mouse anti-His antibody and horseradish peroxidase-conjugated anti-mouse IgG. **(c)** Pseudo-coloured images showing the binding of control samples (bovine serum albumin (BSA) and phosphate buffered saline (PBS)) and different concentrations of purified Spike glycoprotein (5 µg, 10 µg, 10 µg, and 50 µg). The images were generated using the Protein Array Analyzer macro plugin in Image J. **(d)** Pseudo-coloured images showing the binding of four replicates (R1, R2, R3, and R4) of 10 µg purified Spike glycoprotein. The images were generated using the Protein Array Analyzer macro plugin in Image J. **(e)** An individual dot plot showing the average normalized percent relative fluorescence units (RFU) (normalized to positive and negative control) of binding of SARS-CoV-2 Spike glycoprotein to the 95 lectins in the lectin array. Average values were calculated from four lectin array runs (of 10 µg Spike glycoprotein). The lectins with visibly high binding are labelled. **(f)** Lectins that displayed > 100% RFU (after normalizing to positive and negative controls) for binding to SARS-CoV-2 Spike glycoprotein. **(g, h)** Normalized percent RFU of binding to the purified Spike glycoprotein for the lectins predicted to bind to N-core glycans (**g**) and O-core glycans (**h**). Green: Lectins predicted to bind to high mannose glycans; Orange: Lectins predicted to bind to complex as well as high mannose glycans; Yellow: Lectins predicted to bind to complex glycans; Red: Lectins predicted to bind to core-fucosylated glycans.

We performed lectin array analysis of recombinant SARS-Related Coronavirus 2, Wuhan-Hu-1 HexaPro Spike Glycoprotein Ectodomain expressed and purified from BEI NR-53587 pαH Vector transfected Expi293 F™ cultures (Figs. 4a, 4b, 4c, S2 d, S2e). As controls, we used BSA (which is not glycosylated) and PBS, and we did not note any high-intensity fluorescence signals for any of the lectins, barring a faint visible signal for the VVA lectin spots in the latter array (Fig. 4c and Supplementary Data 1). High-intensity fluorescence signals were observed for many of the lectin spots in the arrays incubated with varying amounts (5 to 50 µg) of Spike glycoprotein (Fig. 4c and Supplementary Data 1), indicative of the presence of glycosylation on Spike and optimal assay conditions. Thirty-five of the 95 lectins showed a significant increase in intensity with an increasing amount of Spike glycoprotein, with a Pearson’s correlation coefficient > 0.5; these were mostly lectins with low relative percent binding intensity (< 25%). Most of the lectins that displayed high binding intensities did not show a high positive Pearson’s correlation coefficient, indicating that 5 µg Spike glycoprotein was already sufficient to obtain robust binding and likely past the linear range (Supplementary Data 1).

Figure 4e shows an individual dot plot containing the average values of four replicates of 10 µg Spike glycoprotein (Fig. 4d and Supplementary Data 1) assayed on the lectin array. We found 32 lectins with very high binding intensities to Spike glycoprotein, with binding intensities exceeding that of the positive control in the lectin array (Figs. 4e-4f and Supplementary Data 1). These lectins were also found to display high intensities (more than positive control on average and > 100% at least in two replicates) in the assays performed with different concentrations of Spike glycoprotein (Fig. 4c and Supplementary Data 1). Amongst these, the lectins, GNA, GS-II, NPA, and PHA-L were found to have significantly higher binding intensities than the positive control spots (p < 0.05, paired two-tailed t-test). The top glycans (among the different N- and O-glycans expected in mammalian cells) predicted to be bound by MotifFinder analysis of the lectins are represented in Figure S3, and includes high/oligo-mannose or hybrid N-glycans (predicted to be bound by lectins such as ConA, AMA, BANLEC, BC2LA, GNA, HHA, NPA, and MNA-M) or complex-type N-glycans (predicted to be bound by lectins such as LcHA, ASA, RS-FUC, GS-II, ORYSATA, PHA-E, AMA, PSA, PHA-L), as well as O-glycans and extension sequences (predicted to be bound by lectins such as ACL and SBA). The glycan motifs of these lectins, as predicted by the MotifFinder analysis, are provided in Table S2. The lectin models of these lectins and their binding propensities as predicted by MotifFinder analysis to the comprehensive list of N- and O-glycans in mammalian cells are provided in Supplementary Data 2 and Supplementary Data 1 of Figs. 2 and 3, respectively. There were 35 lectins that bound with lower intensities than the positive control spots, albeit the binding intensities were significantly higher than the negative control spots (p < 0.05, paired two-tailed t-test) (Supplementary Data 1 of Figs. 4d, e). We found 28 lectins that did not bind to the Spike glycoprotein (p > 0.05, paired two-tailed t-test with the negative control) including BC2LCN, BPA, Discoidin I, ECA, GAL1, GAL2, GHA, GS-1, HAA, IRA, Jacalin, LAL, LBA, Lotus, PA-1L, PNA, PPL, PSL1 A, PWA, SHA, SJA, SNA-II, SSA, UEA I, UEA II, VFA, VRA, VVA-M (Supplementary Data 1 of Figs. 4d, e).

### Lectins predicted to bind to N-core glycans

We analyzed the binding of the Spike glycoprotein by the lectins predicted to bind to N-glycan core sequences (Fig. 4g and Supplementary Data 1). We found that the lectins, AAL, AMA, ASA, BANLEC, BC2L-A, CA, CAA, ConA, DSA, GNA, GS-II, HHA, LcHA, UDA, MNA-G, MNA-M, NPA, ORYSATA, PHA-E, PHA-L, PSA, TL, and WGA showed > 50% normalized percent fluorescence intensity to Spike glycoprotein (Fig. 4g). This included lectins with high binding propensities for high/oligo-mannose N-glycan cores such as ASA, BANLEC, BC2L-A, and UDA, as well as lectins with high binding propensities for complex-type N-glycan cores such as PHA-L, DSA, TL, and PHA-E, and lectins with high binding propensities for both high/oligo-mannose and complex-type N-glycan cores such as ConA, GNA, NPA, ORYSATA, AMA, HHA, MNA-G, MNA-M, CA, CAA, GS-II, LcHA, and WGA (Figs. 2c, 4g). The lectins ABA, BC2LCN, LBA, LEA, LTL/Lotus, RPA, SNA-II, and VFA showed low or negligible binding to Spike glycoprotein (Fig. 4g). Considering the predicted binding of PSA to the core fucosylated complex-type N-glycan (Fig. 2c, Table S1), our lectin array results indicate the presence of core-fucosylation on the Spike glycoprotein (Fig. 4g). Further, the absence of binding to RPA (Fig. 4g), which is predicted to bind to a bisecting GlcNAc branch or tri- or tetra-antennary complex-type N-glycan cores (Fig. 2c, Table S1), suggests the presence of biantennary N-glycan cores on the Spike glycoprotein. The high binding to PHA-E (Fig. 4g), which is predicted to bind to a bisecting N-glycan or to biantennary N-glycan terminal hexoses (Fig. 2c, Table S1), might be explained by the presence of extension sequences terminating in hexoses on the Spike glycoprotein.

### Lectins predicted to bind to O-core glycans

We analyzed the binding of the Spike glycoprotein by the lectins predicted to bind to O-glycan core sequences (Figs. 4h and Supplementary Data 1). We found that ACG, ACL, MOA, SBA, and WGA bound with high binding intensities to Spike glycoprotein, whereas AAA, BPA, CNL, CSA, DBA, HAA, Jacalin, PA-IL, PNA, UEA-II, VVA, and WFA bound with relatively low intensities (Fig. 4h). Considering that all these lectins are also predicted to bind to extension sequences (Table S1), this does not necessarily indicate the presence of O-glycans on the Spike glycoprotein of SARS-CoV-2.

To further explore the identity of lectins capable of binding to the O-glycans, if any, on the recombinant Spike glycoprotein expressed from Expi293 F™ cells, we subjected the Spike glycoprotein to beta-elimination for the removal of O-glycans, and then performed lectin array assays (Figures S2a, S2f., S2b). No change in mass was observed upon beta-elimination (Figure S2a, S2f.), as expected, considering the few O-glycosites (such as T323 and S325) that are expected to be occupied by short O-glycans, as per reported literature^35,39,40^. No significant inhibition was found (considering a threshold of > fivefold change and p-value < 0.1 in at least two replicates in a paired t-test) for any of the possible top-binding lectins predicted to bind to O-glycans (ABL, ACL, LSL-N, RCA-60, and SBA (Figure S2c and Supplementary Data 1). Further, similar levels of reduction in binding (as observed for these O-glycan-binding lectins) were also observed for some lectins predicted to bind to N-glycans (Figure S2c), perhaps due to the peeling of N-glycans during the β-elimination reaction.

### Monosaccharide competition assays

**Fig. 5:**
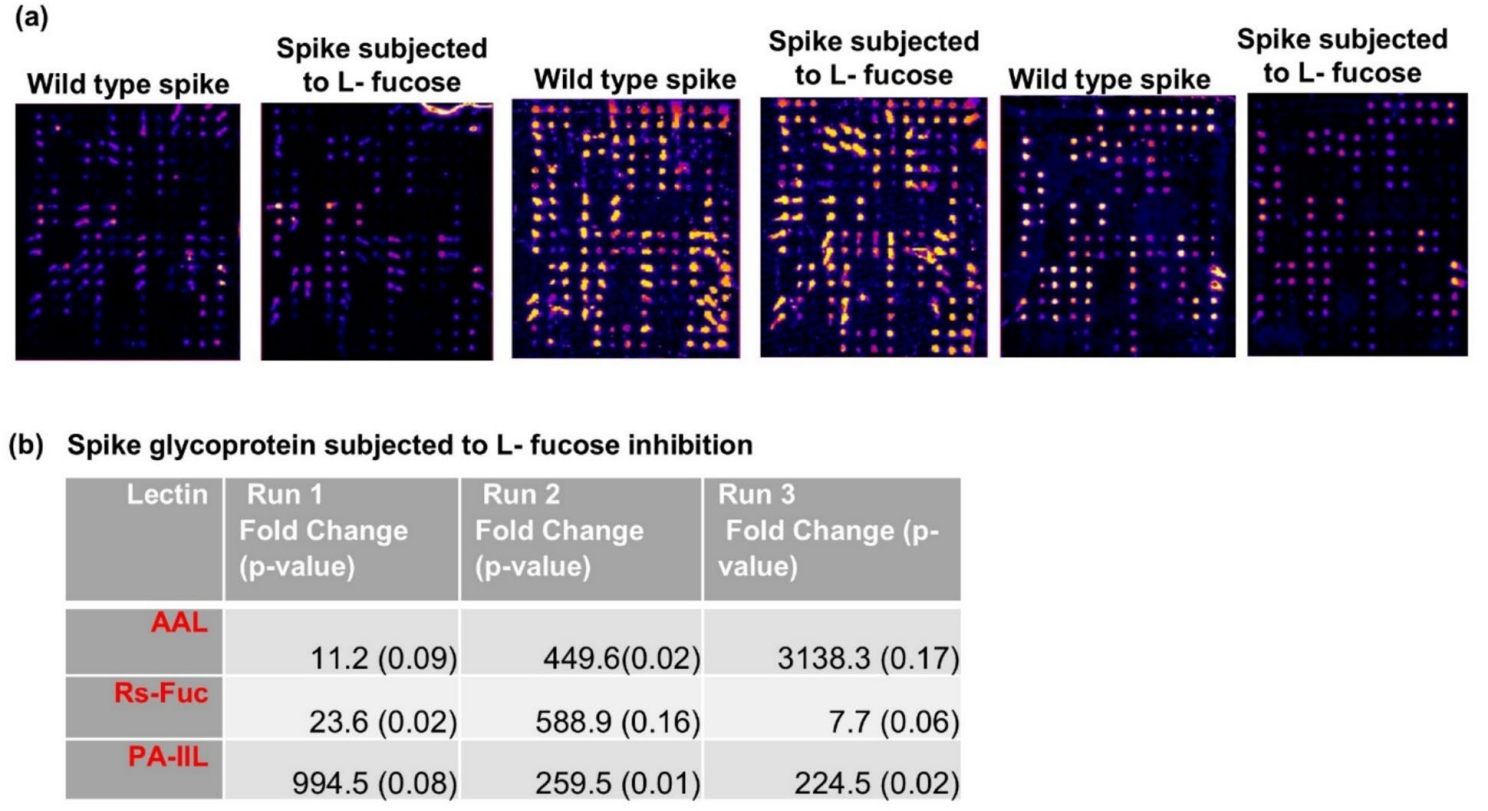
Binding of L-fucose-specific lectins to Spike glycoprotein. **(a)** Pseudo-coloured images showing the binding of Spike glycoprotein (10 µg) in the presence or absence of 100 mM L-fucose. The images were generated using the Protein Array Analyzer macro plugin in Image J. **(b)** Lectins that showed significant inhibition of binding to Spike glycoprotein in the presence of 100 mM L-fucose (n = 3).

We performed lectin array assays of the Spike glycoprotein in the presence of the monosaccharides, 500 mM mannose, 100 mM L-fucose, and 100 mM Neu5 Ac, to assess competitive inhibition, if any, of lectin binding by these monosaccharides (Fig. 5a, Figures S4a, b). We found that the binding of the lectins, PA-IIL, RS-FUC, and AAL, was inhibited by 100 mM L-fucose (considering a threshold of > fivefold change and p-value < 0.1 in at least two replicates in a paired t-test) (Fig. 5b and Supplementary Data 1). Further, no such statistically significant inhibition was observed for these lectins upon incubation with 500 mM mannose or 100 mM sialic acid (Supplementary Data 1), thus validating the L-fucose-specific binding of these lectins to the glycans on the Spike glycoprotein.

No significant inhibition was observed in the binding of any of the mannose and sialic acid binding lectins upon incubation with 500 mM mannose and 100 mM sialic acid, respectively (considering a threshold of > fivefold change and p-value < 0.1 in at least two replicates in a paired t-test), although the N-glycan binding lectins, GS-II and PSA, did show a modest degree of inhibition upon D-mannose competition (Figures S4c, S4 d and Supplementary Data 1). It is possible that these and other mannose and sialic acid binding lectins have low binding affinities for the monosaccharide, which consequently cannot competitively inhibit binding to the more complex glycan motifs on the Spike glycoprotein.

### Lectins binding to the cultured SARS-CoV-2

**Fig. 6:**
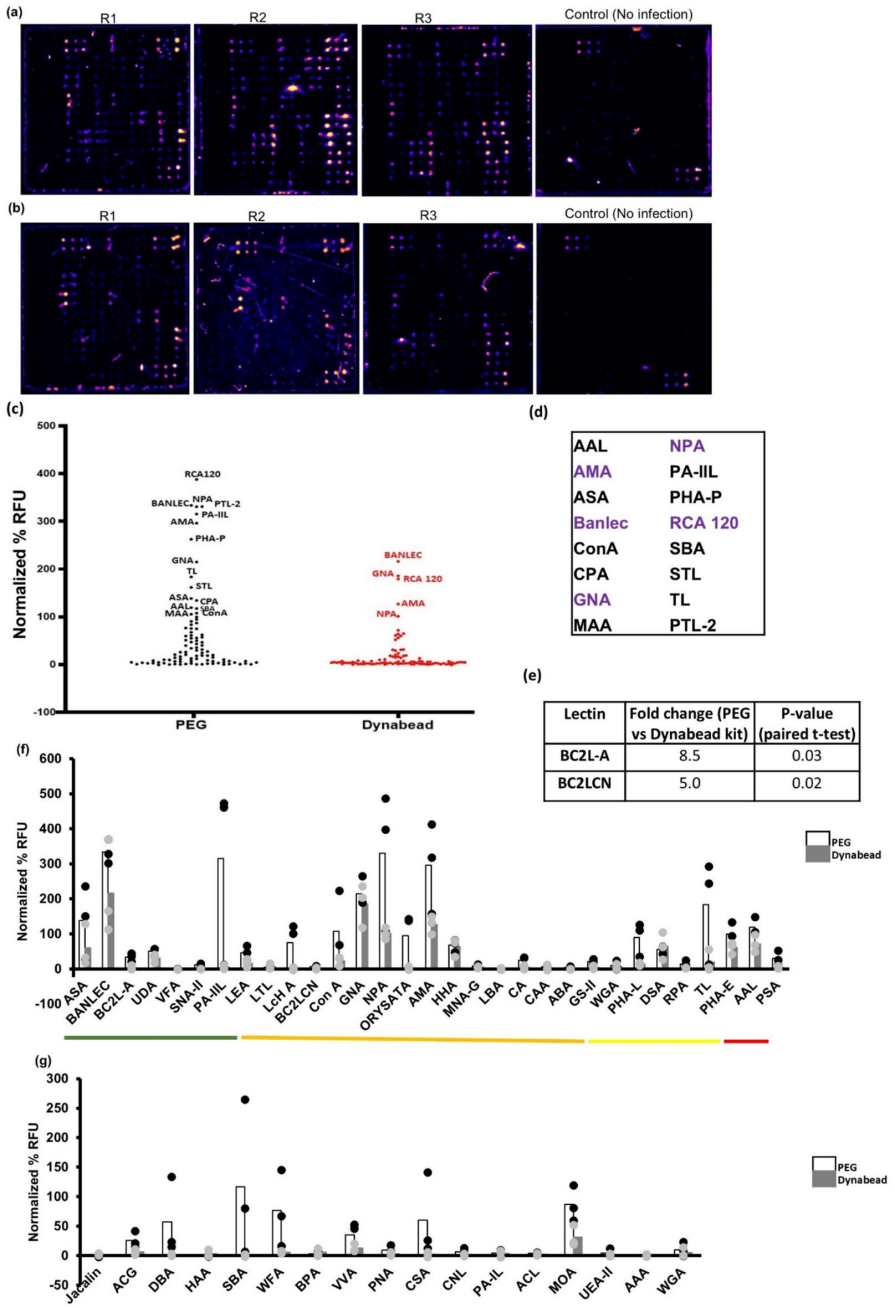
Binding of lectins to SARS-CoV-2 cultured virus: **(a)** Pseudo-coloured images showing the binding of three replicates of SARS-CoV-2 enriched with PEG, and one control (culture supernatant from uninfected Vero E6 cells processed similarly). The images were generated using the Protein Array Analyzer macro plugin in Image J. **(b)** Pseudo-coloured images showing the binding of three replicates of SARS-CoV-2 enriched with Dynabead virus enrichment kit, and one control (culture supernatant from uninfected Vero E6 cells processed similarly). The images were generated using the Protein Array Analyzer macro plugin in Image J. **(c)** An individual dot plot showing the average normalized percent relative fluorescence units (RFU) (normalized to positive and negative control) of binding of SARS-CoV-2 enriched with PEG (in black color) and Dynabead virus enrichment kit (in red color) to the 95 lectins in the lectin array. Average values were calculated from three lectin array runs. The lectins with visibly high binding are labeled. (**d**) Lectins that bound with intensities greater than the positive control spots. The lectins in purple were identified in viruses enriched by PEG and Dynabead virus enrichment kit, and the lectins in black were identified only in viruses enriched by PEG. (**e**) Lectins with > fivefold differences in binding (p < 0.05, two-tailed paired t-test) to virus enriched by PEG versus that enriched by Dynabead virus enrichment kit. **(f,g)** Normalized percent RFU of binding to SARS-CoV-2 for the lectins predicted to bind to N-core glycans (**f**) and O-core glycans (**g**). Green: Lectins predicted to bind to high mannose glycans; Orange: Lectins predicted to bind to complex as well as high mannose glycans; Yellow: Lectins predicted to bind to complex glycans; Red: Lectins predicted to bind to core-fucosylated glycans.

We performed lectin array analysis of Wuhan SARS-CoV-2 virions propagated in Vero E6 cells (Figures S5a, S5b), enriched using centrifugal membrane filters and either PEG or a Dynabeads™ Intact Virus Enrichment kit (Figures S5c, S5 d), inactivated by heat and UV^41^, and biotinylated with biotin-NHS. Enrichment was confirmed by RT-PCR for E-gene (HEX channel) and Orf1ab gene (FAM channel) (Figure S5c, S5 d) by significant differences in the Ct values for the RT-PCR performed with the un-enriched virus and the enriched virus (Figure S5c, S5 d and Supplementary Data 1). For controls for the lectin array, we subjected culture supernatants of uninfected Vero E6 cells to the same enrichment procedures mentioned above. We detected no fluorescence for any of the lectin spots in these controls (Figs. 6a, 6b). High-intensity fluorescence signals were observed for many of the lectin spots in the arrays incubated with SARS-CoV-2 virus enriched either by PEG or by the Dynabeads™ Intact Virus Enrichment kit, indicative of the presence of glycosylation on the SARS-CoV-2 virions.

Figure 6c shows an individual dot plot containing the average values of three replicates each of SARS-CoV-2 enriched either by PEG or by the Dynabeads™ Intact Virus Enrichment kit and assayed on the lectin array. We found many lectins with binding intensities exceeding that of the positive control in the lectin array, and these included AAL, AMA, ASA, BANLEC, Con A, CPA, GNA, MAA, NPA, PA-IIL, PHA-P, PTL-2, RCA-120, SBA, STL, and TL in the case of SARS-CoV-2 virions enriched by PEG, and AMA, BANLEC, GNA, NPA and RCA-120 in the case of SARS-CoV-2 virions enriched by the Dynabeads™ Intact Virus Enrichment kit (Fig. 6c and Supplementary Data 1). The top glycan motif (among the different N- and O-glycans expected in mammalian cells) predicted to be bound by each of these lectins is represented in Figure S3, and includes high/oligo-mannose, or hybrid N-glycans (predicted to be bound by ConA, AMA, BANLEC, GNA, and NPA) or complex-type N-glycans (predicted to be bound by ASA, AMA, RCA-120, PTL-2, PA-IIL, PHA-P, TL, STL, AAL, and MAA), as well as O-glycans and extension sequences (SBA). The glycan motif bound by CPA is unknown; it is reported to haemagglutinate rabbit erythrocytes with inhibition by fetuin, and GalNAc^42,43^.

Overall, we found better lectin binding for SARS-CoV-2 enriched by PEG, with 16 lectins displaying fluorescence intensities exceeding that of the positive control spots in SARS-CoV-2 enriched by PEG compared to only five lectins for SARS-CoV-2 enriched by the Dynabeads™ Intact Virus Enrichment kit, with AMA, BANLEC, GNA, NPA, PA-IIL, PHA-P, PTL-2, and RCA-120 in the former set, and GNA and BANLEC in the latter set displaying significantly higher binding intensities than the positive control spots (p < 0.05, paired two-tailed t-test) (Figs. 6c, 6 d and Supplementary Data 1). We also noted 62 and 26 other lectins in the PEG and Dynabeads™ Intact Virus Enrichment kit sets, respectively, that displayed binding intensities significantly higher than the negative control spots (p < 0.05, paired two-tailed t-test) (Supplementary Data 1), and the remaining lectins did not bind to the Spike glycoprotein (p > 0.05, paired two-tailed t-test with the negative control) (Supplementary Data 1). Interestingly, the glycan binding profiles of the top binding lectins are quite distinct. The five lectins binding to SARS-CoV-2 enriched by the Dynabead kit mainly bind to high/oligo-mannose or hybrid N-glycan cores, with the exception of RCA-120, whose top predicted glycan binder is a galactose terminating biantennary N-glycan (Figure S3). Comparison of the binding intensities of all the lectins in the array for virus enriched by PEG and Dynabeads™ Intact Virus Enrichment kit sets however indicated only two lectins with low to moderate intensities that displayed a statistically significant difference (fold change > 5; p < 0.05, paired two-tailed t-test) (Fig. 6e and Supplementary Data 1).

### Lectins predicted to bind to N-core glycans

We analyzed the binding of SARS-CoV-2 by the lectins predicted to bind to N-glycan core sequences (Fig. 6f and Supplementary Data 1). We found that the lectins, AAL, AMA, ASA, BANLEC, ConA, DSA, GNA, HHA, LcHA, NPA, ORYSATA, PA-IIL, PHA-E, PHA-L, TL, and UDA showed significant binding to SARS-CoV-2 virions enriched by PEG (Fig. 6f). The lectins ABA, BC2L-A, BC2LCN, CA, CAA, GS-II, LBA, LEA, Lotus, MNA-G, MNA-M, PSA, RPA, SNA-II, VFA, and WGA showed low or negligible binding to SARS-CoV-2 virions enriched by PEG or the Dynabeads™ Intact Virus Enrichment kit (Fig. 6f). The absence of binding to PSA is suggestive of the absence of core-fucosylation on the SARS-CoV-2 virions propagated in Vero E6 cells (Fig. 6f).

### Lectins predicted to bind to O-core glycans

We analyzed the binding of the SARS-CoV-2 virions by the lectins predicted to bind to O-glycan core sequences (Fig. 6g and Supplementary Data 1). We found that only CSA, DBA, MOA, SBA, and WFA bound with high (> 50% normalized %RFU) binding intensities to SARS-CoV-2 virions enriched by PEG (Fig. 6g). Considering that all these lectins are also predicted to bind to extension sequences (Table S1), this does not necessarily indicate the presence of O-glycans on SARS-CoV-2.

### Lectins binding to recombinant Spike glycoprotein and SARS-CoV-2 virions

We analyzed the predicted glycan binding profiles of lectins that bound with high intensities to the recombinant Spike glycoprotein or to the SARS-CoV-2 virions (ABL, ACL, RCA 120, AMA, CALSEPA, GAL3, GS-II, PALa, CA, HHA, PHA-L, PA-IIL, MNA-M, BC2L-A, STL, LSL-N, GRFT, PSA, RS-FUC, PHA-E, CPA, LENTIL, RCA 60, GNA, ORYSATA, LcH A, PHA-P, PTL-2, MAA, Con A, BANLEC, TL, AAL, NPA, ASA, SBA) (Table S3 and Supplementary Data 1), and found that some glycan motifs were significantly predicted to be bound by these lectins (Figure S6a and Supplementary Data 1). Considered together, we predict the glycans on the recombinant Spike glycoprotein and SARS-CoV-2 virions to contain high mannose/hybrid N-glycans with terminal mannose residues, α1–6 core fucosylated N-glycans with terminal GlcNAc residues, and complex glycans with Lewis A, Lewis B, Lewis X, Lewis Y, and Blood group H structures on type-1 or type-2 extension sequences (Figure S6a).

A comparison of lectin binding by the recombinant Spike glycoprotein versus SARS-CoV-2 virions indicated some lectins that significantly differed in their binding (p < 0.05, unpaired t-test) (Table S4, and Supplementary Data 1). Analysis of the glycan motifs predicted to be differentially bound by these lectins vis-à-vis the remaining lectins indicated that N-glycans terminating in GlcNAc and type 1 extension sequences and several O-glycan cores were significantly enriched (p < 0.05, unpaired t-test) (Figure S6b and Supplementary Data 1). The differential binding by lectins to the Spike glycoprotein expressed from Expi293 F™ cells, and to the SARS-CoV-2 virions propagated from Vero E6 cells could stem from differential expression of the enzymes involved in the synthesis of these glycans or differential processing of the glycoprotein.

## Discussion

The SARS-CoV-2 viral envelope is covered with highly glycosylated Spike protein, essential in viral binding and entry into the host cells^44,45^. In the therapeutic field, targeting glycosylation offers possibilities for inhibiting viral infections such as COVID-19. Lectins are, therefore, lead molecules for developing new antiviral agents based on the ability to inhibit viral entry in the host cell. Here, we have screened 95 different lectins from different sources (animals (12), plants (65), fungi (10), microbes (5), slime moulds (2), and red alga (1)) on a commercial lectin array for their potential to bind to the SARS-CoV-2 Spike glycoprotein expressed from Expi293 F™ cells and the SARS-CoV-2 virions propagated in Vero E6 cells. Although a few studies have recently used lectin arrays – Senicar et al. to distinguish the lectin profile of SARS-CoV-2 negative and SARS-CoV-2 positive nasopharyngeal swabs^46^, Wuo et al. to understand the glycome of SARS-CoV-2 Spike glycoprotein in relation to antibody neutralization^47^, Hoffman et al. to identify mammalian lectins that bind to the RBD of Spike^48^, and Zheng et al. to understand the changes in the glycome associated with different variants of concern of Spike^31^, our study represents a notable expansion compared to the previous studies, with a more detailed examination of lectin-binding specificities associated with SARS-CoV-2, as predicted from the available glycan array data on Carbogrove^38^ (68 lectins out of 95 lectins) using the MotifFinder tool^49^ against N-core, O-core, N-extension, and O-extension glycans.

There are several studies that have examined the site-specific glycosylation profile of SARS-CoV-2 Spike glycoprotein using mass spectrometry^9,14,34,50–54^ (Table S5 and Supplementary Data 1). The glycan shield of SARS-CoV-2 is accepted to be sparse and mainly dominated by complex-type glycans as compared to other viral glycan shields^52,55–57^. Out of 22 N-linked glycosylation, 8 sites contain a considerable population of oligomannose-type glycans, while 14 sites are dominantly covered with complex-type glycan^9,34^. Whereas site-specific glycosylation and glycan composition data are abundantly present for the SARS-CoV-2 Spike glycoprotein, glycan linkage data is absent. Here, we have used a lectin panel to address this gap, and our results indicate that high mannose/hybrid N-glycans with terminal mannose residues, α1–6 core fucosylated N-glycans with terminal GlcNAc residues, and complex glycans with Lewis A, Lewis B, Lewis X, Lewis Y, and Blood group H structures on type-1 or type-2 extension sequences are present on the recombinant Wuhan SARS-CoV-2 Spike glycoprotein and on Wuhan SARS-CoV-2 virions.

Our study indicates a few differences in the glycan profiles of lectins binding to recombinant (Wuhan) Spike glycoprotein and (Wuhan) SARS-CoV-2 virions—N-glycans terminating in GlcNAc and type 1 extension sequences and several O-glycan cores. These differences may stem from the different cell types (Expi293 F™ vs Vero E6)^40^, different processing routes (recombinant glycoprotein ectodomain secretion vs virus budding), different glycoconjugate moieties (Spike glycoprotein vs Spike and other glycoproteins on the intact virions; for instance, Griffithsin inhibited hACE2-mediated direct infection better when the coronaviral M protein was present than when it was absent, which may explain why it inhibits authentic SARS-CoV-2 better than pseudotyped viruses, which do not contain M^58^), different degrees of enrichment and resulting contamination with other glycoproteins secreted by the cells or present in the serum-free culture medium (due to the different principles of enrichment, i.e., molecular exclusion by the non-ionic polymer PEG^59^ and charge-based enrichment by the Dynabead kit), and the effect of enrichment conditions on the Spike glycans (effect of exposure to acidic pH during enrichment using the Dynabead kit^60^, and longer processing times with the enrichment method by PEG). Previous studies in the literature have also indicated that Spike glycosylation can vary according to the lab (and researchers) performing the study, expression cell lines, and the sample being studied (glycoprotein or pseudovirus or clinical sample), although overall consensus has also been demonstrated between the glycan profiles obtained for recombinant stabilized Spike (2P) glycoprotein preparations from different laboratories and viral derived Spike glycoprotein^34,53^. Variable low-occupancy O-glycosylation has been previously reported in the Spike glycoprotein^39,40,50,54,61^. Reported glycans include Tn antigen, core-1, sialylated core-1, core-2, and sialylated core-2 glycans^50,62^. While our assays did indicate high intensities of binding by ACL^63^ (predicted to bind to core-1 and core-2 O-glycans) and SBA (predicted to bind to Tn, core-5, and core-7 O-glycans), we observed no significant change upon beta-elimination. ACL and SBA^63^ were also predicted to bind to GalNAc in N- glycan extension sequences; hence, it is possible that these lectins actually bound to N-glycans on the Spike glycoprotein.

The limitations of our study include the absence of anti-carbohydrate antibodies (which are more specific and more sensitive than lectins) in this commercial array, the absence of a readout indicating functional activity for some of the lectins (that did not bind to PAA-monosaccharides or to our samples), the absence of glycan array data for some of the lectins in the array and the lack of information relating to the exact sources of the lectins in the array (raising the possibility that the publically available glycan array data does not correspond to the same sources of lectins used in the commercial lectin array and, consequently, does not accurately reflect the binding profile of the lectins immobilized on the array), and the inability to quantify the relative abundance of different glycosylation features (such as sialylation versus fucosylation) due to the variable affinities and specificities of different lectins for the multiple glycans on the Spike glycoprotein, which is further complicated by multivalent presentation. Our study also only employed beta-elimination to probe the effect of O-glycan removal, and did not employ treatment with specific enzymes (such as PNGase, endo-H, sialidases, etc.), and we did not observe any competitive inhibition by monosaccharides for mannose and sialic acid binding lectins, raising questions about the specificity of binding by these lectins, albeit this might also be explained by low binding affinity for the monosaccharide. Considering the differences we observe in the glycan profiles of recombinant (Wuhan) Spike glycoprotein and (Wuhan) SARS-CoV-2 virions enriched by two different methods, which could stem from the different cell types, it is possible, too, that the glycosylation status of the virus in the infection setting might be different from what we observe here.

To summarize, we assessed the SARS-CoV-2 binding ability of 95 lectins (on the lectin array). We predicted the glycan binding profiles of 68 of these 95 lectins, for which publically available glycan array data was available, and found that 67 of these 68 lectins were predicted to bind to either N-glycans or O-glycans or both (Figure S7a and Supplementary Data 1). We also found that 32 of the 95 lectins on the array bound with high intensity to the recombinant Spike glycoprotein, and four of the 95 lectins (CPA, MAA, PA-IIL, and STL) that did not bind with high intensity to the recombinant Spike glycoprotein bound to PEG-enriched SARS-CoV-2 virions (Figure S7b,c and Supplementary Data 1); of these 32 lectins, seven lectins also bound to PEG-enriched SARS-CoV-2 virions but not to Dynabead kit-enriched SARS-CoV-2 virions. Importantly, five of these 32 lectins (AMA, BANLEC, GNA, NPA, and RCA-120) that bound with high intensity to the recombinant Spike glycoprotein also bound with high intensities to PEG-enriched SARS-CoV-2 virions and Dynabead kit-enriched SARS-CoV-2 virions and represent the five most promising lectins identified in our study (Figure S7b,c and Supplementary Data 1).

A significant number of lectins bind to the recombinant Spike glycoprotein expressed from Expi293 cells in our study. The 32 lectins that bound with high intensity to the recombinant Spike glycoprotein include lectins that bind to oligomannose/hybrid and complex-type N-glycans, extension sequences, and O-glycans. They include BANLEC, Lentil, GRFT, HHA, NPA, GNA, MNA-M, and Gal3, which have been previously suggested to have anti-SARS-CoV-2 or anti-SARS-CoV activity^24,25,30,64–66^, and a few lectins, GS-II, PHA-E, and PHA-L, which were previously shown to interact with the nasopharyngeal samples of SARS-CoV-2-positive patients^46^. They also include the lectins, AAL, ABL, ACL, AMA, ASA, BC2L-A, CA, CALSEPA, ConA, LcHA, LSL-N, ORYSATA, PALa, PHA-P, PSA, PTL-2, RCA-60, RCA-120, RS-FUC, SBA, and TL. A comparison with a published article describing a comprehensive study of HEK293 glycosylation using lectins as well as mass spectrometry^67^indicates that the lectins identified in our study are likely only a subset of the lectins that can bind to HEK293 cells. For instance, the lectins DSA, UEA-1, WGA, WFA, and LEA bind to HEK293 cells (as reported by Huang et al^67^.) but do not bind to Spike glycoprotein in our study, suggesting that the Spike glycoprotein might not merely have all the glycan motifs that are biosynthetically possible on Expi293 cells.

We classified the panel of 36 lectins that bound with high intensities to the recombinant SARS-CoV-2 Spike glycoprotein and/or the SARS-CoV-2 virions as per the Lectin Frontier DataBase (LfDB)^68,69^ and found that these lectins belonged to the Agglutinin, B lectin, Chitin bind 1, Fungal lectin, FB lectin, Gal bind lectin, Jacalin, Lectin legb, PA-IIL, and Ricin B lectin families, and seven lectins were unclassified by this database (Table S6 and Supplementary Data 1). Considering the number of SARS-CoV-2 binding lectins classified under each of these families, the Lectin legb (Legume lectin), FB lectin (Fungal Fruit Body lectin), and B lectin (Bulb lectin) families represented the most promising families for interaction with SARS-CoV-2. Analyzing the five most promising lectins that bound with high intensity to the recombinant Spike glycoprotein as well as to PEG-enriched SARS-CoV-2 virions and Dynabead kit-enriched SARS-CoV-2 virions (AMA: B lectin family, BANLEC: Jacalin family, GNA: B lectin family, NPA: B lectin family, and RCA-120: Ricin B lectin family), we observed that whereas AMA, BANLEC, GNA, and NPA are predicted to preferentially recognize high/oligo-mannose/hybrid N-glycans, RCA-120 is predicted to bind to complex-type N-glycans terminating in LacNAc.

Identifying lectins with specific binding profiles to SARS-CoV-2 holds promising implications for therapeutic development and diagnostic assays. Viral proteins are generally covered with under-processed high mannose and hybrid glycans)^55,70^, resulting in the use of mannose-binding lectins as antiviral agents^71^. In contrast, the SARS-CoV-2 Spike glycoprotein is also decorated with complex-type glycans^34^. Further, the RBD (residues 320 to 527) of SARS-CoV-2 Spike glycoprotein that binds to ACE2 to facilitate virus entry contains two N-glycosylation sites, N331 and N343, both of which are occupied by highly complex type N-glycans (as determined by mass spectrometry analyses of the recombinant Spike glycoprotein as well as viral-derived Spike glycoprotein^33,34^), and two O-glycosylation sites with low occupancy, T323 and S325 (3.38% and 0.5%, respectively). Lectins specific for complex N-glycans (such as RCA-120) might, therefore, be more likely to interact with the RBD and inhibit ACE2 binding and subsequent viral entry. However, for antiviral therapeutic applications, it would also be important to consider the nature of the native host cell surface glycans. Cell surfaces in human tissues are predominantly decorated with complex glycans^72^, and the respiratory tract, which is infected with the SARS-CoV-2 virions, has a variety of sialylated complex glycans^73^. In such a scenario, mannose-binding lectins such as AMA, BANLEC, GNA, and NPA seem more likely to bind to SARS-CoV-2 virions in the physiological milieu. However, lectins that bind to complex N-glycans might also be able to bind to SARS-CoV-2 virions, despite the overwhelming presence of complex glycans on host cell surfaces in the physiological milieu, provided that these lectins have sufficiently narrow-specificity glycan binding profiles that they are able to discriminate between the glycans on the virus and uninfected host cells in the vicinity. In the event that the lectins successfully bind to the virus in the physiological setting with minimal binding to host cell receptors, it would also be important to assess whether binding to SARS-CoV-2 Spike actually results in inhibited ACE2 binding and virus entry under physiological conditions. The lectins identified in this study could be subjected to such studies in the future to explore their potential to serve as anti-viral agents or markers for virus detection.

## Material and methods

The study protocols were approved by the Institutional Biosafety Committee (CSIR/IMTECH/IBSC/2020/J18), and all experiments were performed in accordance with their guidelines. This study does not involve any human participants or human tissue samples. All methods were carried out in accordance with relevant guidelines and regulations.

### Analysis of glycan-specificities of lectins using glycan array data

#### Building lectin models

We analyzed 68 lectins (out of the 95 lectins on the lectin array, for which glycan array datasets were available publicly) for their glycan-binding specificities. Glycan array datasheets of each lectin at different concentrations from different protein sources and different data sources were put together by arranging the files “AllBindingData.csv, AllDataInfo.csv, and AllGlycanInfo.csv” downloaded from the API of the CarboGrove database (https://carbogrove.org/api/home.php). Downloaded datasheets were named Lectinname_Proteinsource_concentration_datasource_DATA and used to build an automated lectin model using MotifFinder v2.2.5 ^38,49,74^ at default settings with the default glycan motif list. The lectin model provided as output, the specificities and fine-specificities of the lectin to different glycan motifs.

### In silico generation of glycans

Four different glycan lists containing N-core glycans, O-core glycans, N-extension glycans, and O-extension glycan, were generated in silico as follows. The N-core glycans were listed manually from N-glycan biosynthesis principles^75^ and contained 24 glycans, which included high mannose glycans, complex glycans, and hybrid glycans, with options of core-fucose, bisecting GlcNAc, and GlcNAc-substitution on Mannose. The O-core glycan list contained 10 glycans, i.e., core 1 to 8, Tn antigen, and sTn antigen, and was listed manually^76,77^. The N-extension glycan list contained 144 glycans with different extensions such as LeA, LeB, LeX, LeY, and others, and was generated in silico using MotifFinder on the biantennary glycan scaffold. The O-extension glycan list contained 121 glycans and was listed manually^76^.

### Predicting the propensity of lectins to bind to the generated glycans

The lectin models (built using MotifFinder) were used as input to predict each lectin’s propensity to bind the list of generated glycans (N-core glycan, O-core glycans, N-glycan extension sequences, and O-glycan extension sequences) using the “Predict Model” tool of MotifFinder software. The output obtained was predicted binding intensities of each lectin to N-core glycan, O-core glycans, N-glycan extension sequences, and O-glycan extension sequences, and this was used to generate a “predicted binding” heat map (http://www.heatmapper.ca/expression/) by using average linkage clustering and Euclidean distance measurement methods. Clustering was applied to rows as well as columns.

### Chemicals and reagents

The Lectin array-95 kit was purchased from Ray Biotech. Glycoprofile β-Elimination kit, monosaccharides, and bovine serum albumin (BSA) were purchased from Sigma. Zeba spin desalting columns were purchased from Thermo Scientific. Plasmid Modified pαH Vector Containing the SARS-Related Coronavirus 2, Wuhan-Hu-1 HexaPro Spike Glycoprotein Ectodomain (Genbank Accession: MN908947^78,79^) was from BEI resources (NR-53587). PAA-Biotin, PAA-Biotin-Mannose, PAA-Biotin-L-Fucose, and PAA-Biotin-Sialic acid were purchased from Glycotech.

### Expression and purification of recombinant Spike glycoprotein

The SARS-CoV-2 Wuhan-Hu-1 HexaPro Spike Glycoprotein Ectodomain was produced in Expi293 F™ cells. The cells were transiently transfected with Modified pαH Vector Containing the SARS-Related Coronavirus 2, Wuhan-Hu-1 HexaPro Spike Glycoprotein Ectodomain, NR-53587 (BEI) by polyethyleneimine at the density of ~ 3 × 10^6^ cells/ml and the culture supernatant was harvested four days post-transfection. The supernatant was centrifuged at 4000 rpm, followed by filtration through a 0.22 µm filter to remove cell debris. The culture supernatant was passed through the nickel ion affinity resin (Thermo Scientific) at 4 °C and bound protein was eluted from the Ni–NTA column using 500 mM imidazole in PBS buffer at pH 7.4. The eluted protein was extensively dialyzed against phosphate-buffered saline using a 10 kDa (MWCO) dialysis membrane. The purity of purified protein samples was assessed by SDS-PAGE.

### Beta-elimination of Spike glycoprotein

Spike glycoprotein (30 μg) was dialyzed against water using a Zeba spin desalting column and then denatured at 70 °C for 30 min. The β-elimination reagent mixture (mixture of sodium hydroxide solution and β-elimination reagent in the ratio provided with the Glycoprofile β-Elimination kit) was added to the protein sample in a volume equal to 20% of the protein sample and incubated at 4 °C for 18 h. The Spike protein was separated from the released O-glycans following β-elimination by passing the reaction mixture through centrifugal membrane filters (MWCO 10 kDa) after neutralizing the mixture (pH 6–8). The Spike protein was then lyophilized, biotinylated, and used for the lectin array assays.

### Cell culture and SARS-CoV-2 propagation

Vero E6 cells were obtained from the National Centre for Cell Science (NCCS) at passage number 12 and cultured in Dulbecco’s minimum essential medium (DMEM) supplemented with 5% fetal bovine serum (FBS) and 0.1 mg/ml Penicillin–Streptomycin. Cells were maintained in T25 cm^2^ flasks at 37 °C with 5% CO₂ until reaching 90% confluency. The SARS-CoV-2 Wuhan strain used in this study was previously isolated and reported (GISAID accession number EPI_ISL_11450498)^80^. It was propagated through three passages in Vero E6 cells, and stored at −80 °C in DMEM supplemented with 5% FBS. This virus stock was used in all the experiments.

### Virus Infection

Vero E6 cells at 90% confluency were infected with SARS-CoV-2 at a volume of 250 µl for one hour at 37 °C. Control cells were treated with an equivalent volume of media. The infection was confirmed by plaque assay (Figures S5a, S5b). Following infection, cells were maintained in DMEM without FBS at 37 °C for 48 h, with daily observation for cytopathic effects such as cell aggregation, rounding of cells, and typically complete detachment of cells from the monolayer on the second day.

### Virus harvest and enrichment

At 48 h post-infection, the virus-containing supernatant was harvested by centrifugation at 400x*g* for 10 min. The resultant supernatant was collected and stored at −80 °C until further analysis. Quantitative analysis was performed using reverse transcription-polymerase chain reaction (RT-PCR) with a GCC Biotech kit.

The harvested supernatant was initially concentrated using an Amicon centrifugal membrane filter with MWCO 30 kDa^81^. Subsequently, virus enrichment was performed using either polyethylene glycol (PEG) precipitation or a Dynabead kit.

### PEG enrichment method

PEG-8000 (80 g), sourced from Sigma Aldrich (Catalog no. 25322–68-3), was dissolved in MilliQ water of type 1. To this solution, 14 g of NaCl was added, followed by the addition of 20 ml of 10X PBS (1X PBS: 137 mM NaCl, 10 mM phosphate, 2.7 mM KCl; pH 7.4) to achieve a final volume of 200 ml. As a result, the resulting solution had a stock concentration of 40% (w/v) PEG and 1.2 M NaCl, forming the PEG concentrator. The concentrated supernatant was mixed with the PEG concentrator at a ratio of 1:3 (v/v) and then incubated on a shaker at 60 rpm at 4 °C overnight. Subsequently, the mixture underwent centrifugation at 1600x*g* for 60 min at 4 °C. Following centrifugation, the supernatant was decanted, and the pellet was reconstituted in 1X PBS for RT-PCR analysis and further use^82^.

### Dynabead intact virus ènrichment kit

The concentrated supernatant was enriched using the Dynabeads™ Intact Virus Enrichment kit (Invitrogen Catalog no. 10700D) as per the instructions^83^. Briefly, the viral supernatant was mixed with Dyna magnetic beads (20 µl) and subjected to a 20-min-incubation on a roller. Following this, the mixture was placed on a magnetic stand for 1 min to remove the supernatant. The beads underwent three washes with 1X PBS. Subsequently, a releasing buffer (50 mM citric acid, 50 mM Na-phosphate, pH 4) was added to liberate the virus from the beads, followed by another 20-min-incubation on a roller. The resulting supernatant was collected and further processed through a 30 K MWCO centrifugal filter (Amicon) for buffer exchange, ultimately yielding the virus in 1X PBS, which was subjected to RT-PCR analysis.

### RT-PCR

RNA isolation from samples was performed using a GCC Biotech RNA Isolation kit, followed by reverse transcription with a DiAGSURE nCoV-19 Detection Assay kit. The resulting cDNA was amplified using primers targeting the E-gene (HEX) and ORF1ab gene (FAM). A cut-off value of Ct ≤ 36 was used for detection.

### Virus inactivation

To enable lectin array analysis outside of biosafety level 3 (BSL3) facilities, virus aliquots underwent UVC treatment for 10 min, followed by heat treatment at 55 °C for 20 min. This treatment has been previously shown to preserve viral glycosylation while ensuring inactivation^41^.

### Biotinylation of spike glycoprotein/SARS-CoV-2 virus

Recombinant Spike glycoprotein was biotinylated before application to the lectin array slides according to the manufacturer’s instructions. Briefly, 30–40 µg spike glycoprotein (in PBS) was labeled with biotin-NHS using the 1 × labeling reagent provided with the kit and incubated for 30 min at room temperature with gentle rocking. The reaction was then stopped using a 3 µl stop solution, the protein was dialyzed extensively against PBS, and the concentration was estimated using the absorbance at 280 nm. SARS-CoV-2 virus, obtained after enrichment and inactivation, was biotinylated by 3.3 µl 1X labeling reagent as per the kit instruction for labeling cells. Like the Spike protein, it was incubated for 30 min, and the reaction was stopped using the stop solution and the virus further dialyzed against PBS.

### Lectin array assays

The lectin binding profiles of recombinant Spike glycoprotein and cultured SARS-CoV-2 virus were detected using the label-based approach provided with the Ray Biotech Lectin Array 95 kit (cat# GA-Lectin-95). Briefly, the lectin array slide was first blocked by incubation with sample diluent at room temperature for 30 min, then 10 µg of biotinylated Spike glycoprotein or 100 µl biotinylated SARS-CoV-2 virus in PBS was incubated on the lectin slide at 4 °C overnight and washed multiple times (10–12 times for 5 min each) using the provided wash buffers. The reaction was then incubated with a Cy3 equivalent dye-streptavidin conjugate in the dark at RT for 1 h and washed multiple times. The slide was then dried, and the signal was visualized using the Cy3 detection settings on a Typhoon Molecular Imager (Cytiva Life Sciences). 1 µg of PAA-Biotin, PAA-Biotin-α-D-Mannose, PAA-Biotin-α-L-Fucose, and PAA-Biotin-α-Neu5Ac were incubated on blocked lectin array slides in the same manner as mentioned above for the initial characterization of the lectin array. For competitive sugar inhibition assays set up to analyze the effect of different sugars on the lectin binding to Spike glycoprotein, biotinylated Spike was pre-incubated with 500 mM D-mannose, 100 mM L-fucose, or 100 mM sialic acid (pH 7) at 4 °C for one hour before application to the blocked lectin array slide. The rest of the steps were the same as described above.

### Densitometry and quantification of lectin array results

The spot intensity of each lectin in the fluorescence scan image obtained from the Typhoon Molecular Imager was determined by using the Protein Array Analyser toolset^84^ under the Macros plugin of the Image J software^85^. Spot intensities were calculated with linear background subtraction (radius of rolling 2D ball: 25). In the case of visible artifacts or misalignment of the spot intensities with the grid in the Protein Array Analyser toolset, individual spot intensities were quantified manually using the same radius of measurement. Spot intensities thus calculated were imported into the GA-Lectin-95 Excel sheet provided with the lectin array kit to sort and map spot intensities to the respective lectins. These mapped intensities were then normalized with respect to the positive and negative controls and, hence, represented as normalized percent relative fluorescence units (RFU). In the case of Spike subjected to β-elimination or competition with monosaccharides, differences in lectin binding compared to the untreated Spike were analyzed by calculating the fold change and performing a paired t-test.

## Supplementary Information

Supplementary Information 1.

Supplementary Information 2.

Supplementary Information 3.

## Data Availability

All source data behind the graphs in the manuscript are being provided in Supplementary Data 1 and all lectin models used in the study are available in Supplementary Data 2. Other data associated with the study are available from the corresponding authors upon reasonable request.
