# Supplementary material for: Identification of SARS-CoV-2-binding lectins on a commercial lectin array
Source: Sci Rep. 2025 Jul 1;15:21687. doi: 10.1038/s41598-025-01903-5 (PMC12217048; doi:10.1038/s41598-025-01903-5)
Supplement: Supplementary file 3 — Supplementary Information 3. [file 41598_2025_1903_MOESM3_ESM.docx]

**Supplementary Information**

**Identification of SARS-CoV-2-binding lectins on a commercial lectin array**

**Neetu^1,2,#^, Shimona Ahlawat^1,2,#^, Rathina Delipan^1^, Rajesh P. Ringe^1^, Alka Rao^1,2,3*^ and T.N.C. Ramya^1,2,*^**

*^1^CSIR- Institute of Microbial Technology, Sector 39-A, Chandigarh 160036, INDIA*

*^2^Academy of Scientific & Innovative Research (AcSIR), Ghaziabad, Uttar Pradesh 201002, INDIA*

*^3^ Present address: Food Safety and Standards Authority of India (FSSAI), New Delhi 110002, India*

*^#^These authors contributed equally to the work and should be considered joint first authors*

**Corresponding authors’ e-mail:* [*ramya@imtech.res.in*](mailto:ramya@imtech.res.in)*,* [*raoalka@imtech.res.in*](mailto:raoalka@imtech.res.in)

**Running Title:** Lectins that bind to SARS-CoV-2 Spike

**Keywords:** SARS-CoV-2, COVID-19, lectins, Spike, glycosylation, lectin array, O-glycans

**This supplementary information .pdf file contains seven supplementary figures, S1-S7, and six supplementary tables, S1-S6.**

**A Supplementary Data 1 .xlsx file containing all the source data behind the graphs in the manuscript and a Supplementary Data 2 zipped folder of all the lectin models described in the paper also accompany this manuscript as supplementary data.**

**Figure S1**

**(a)**


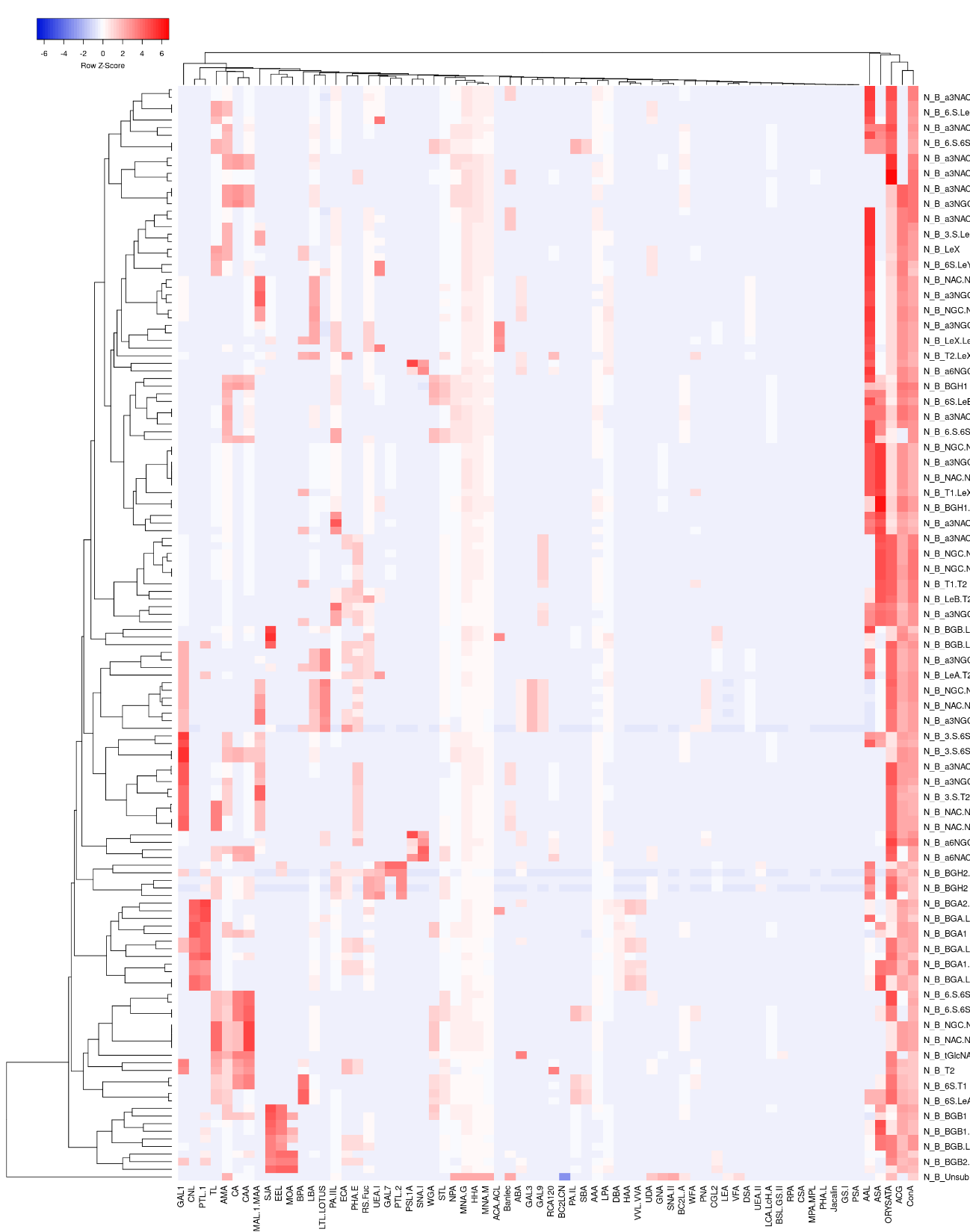

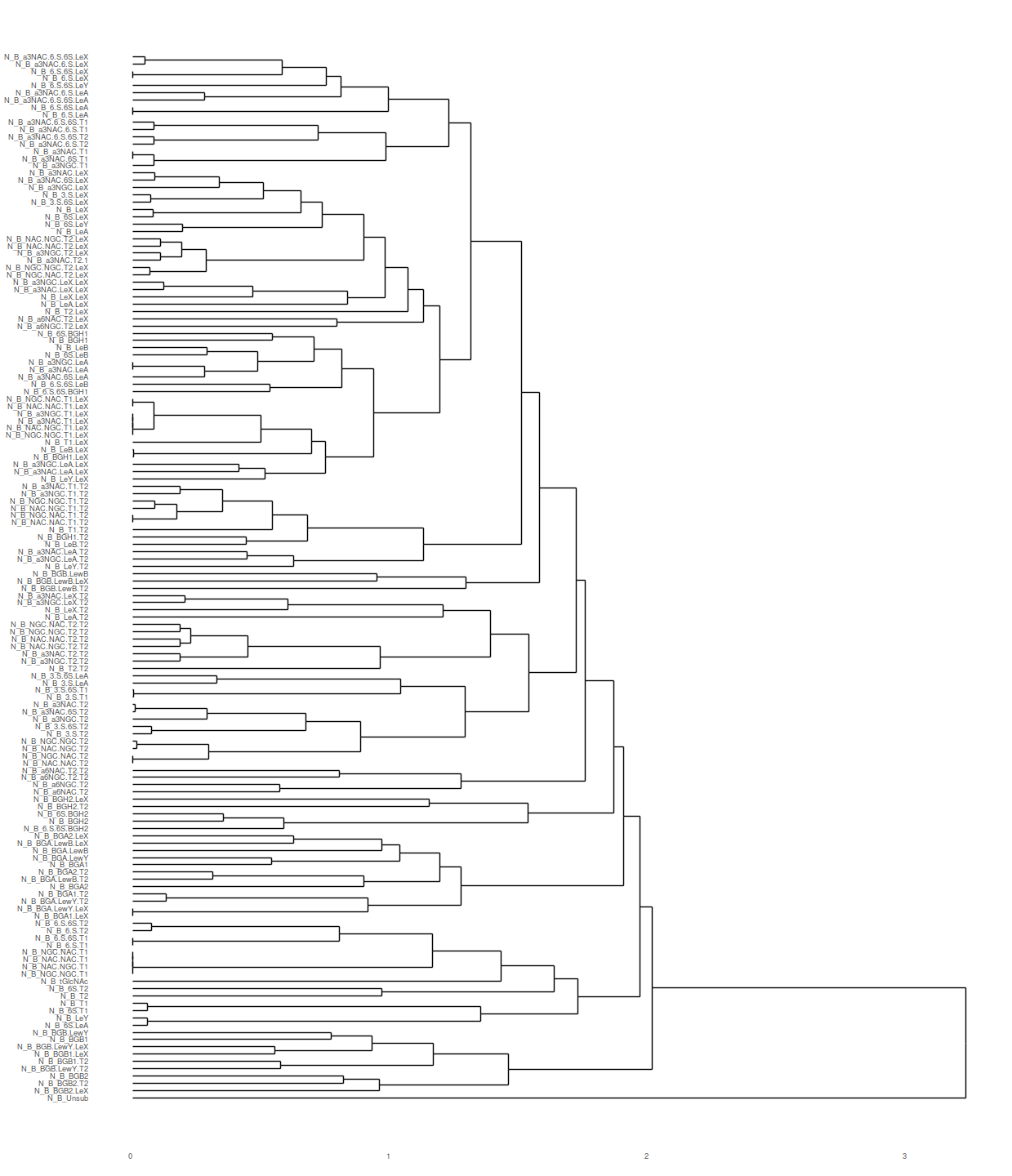

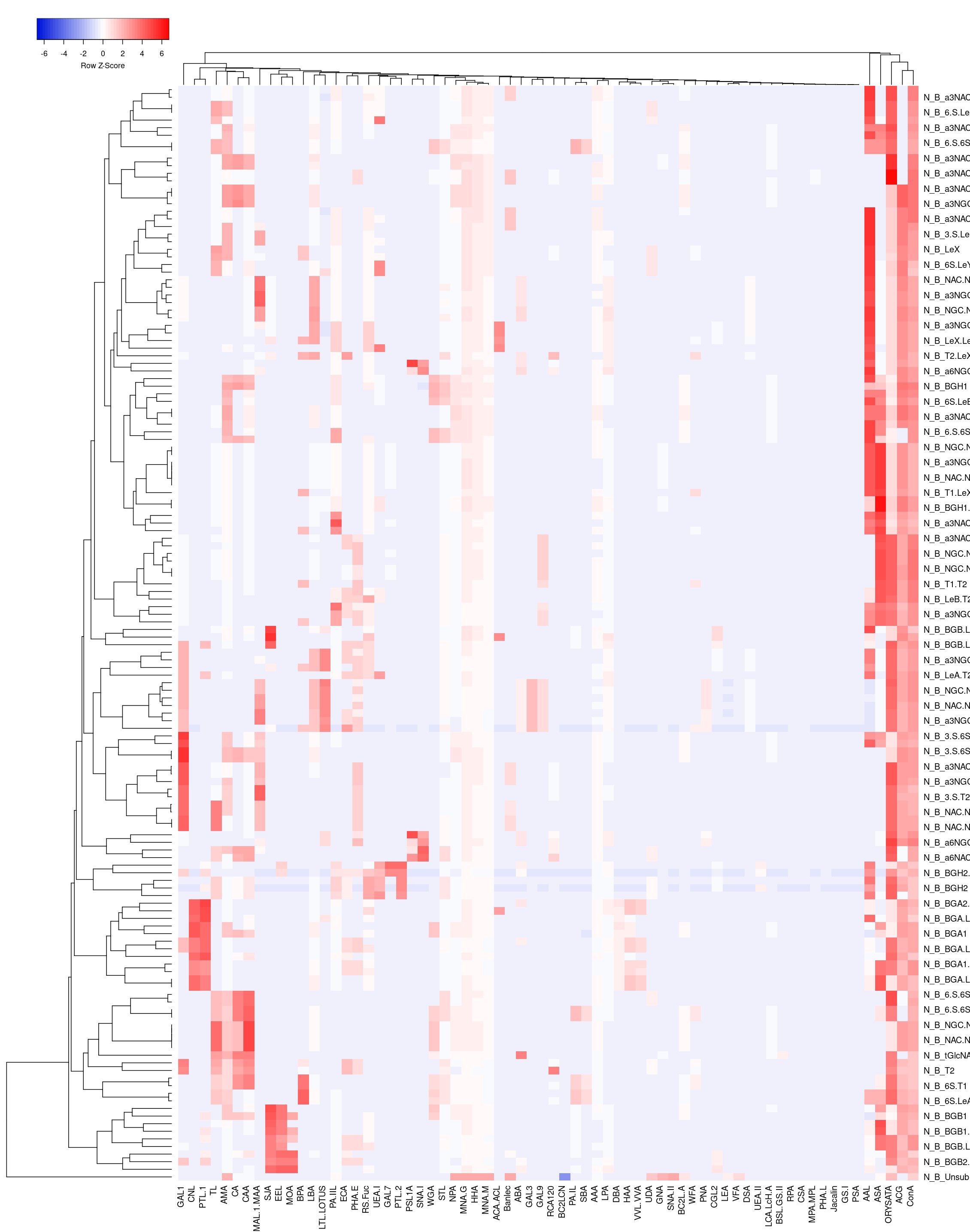

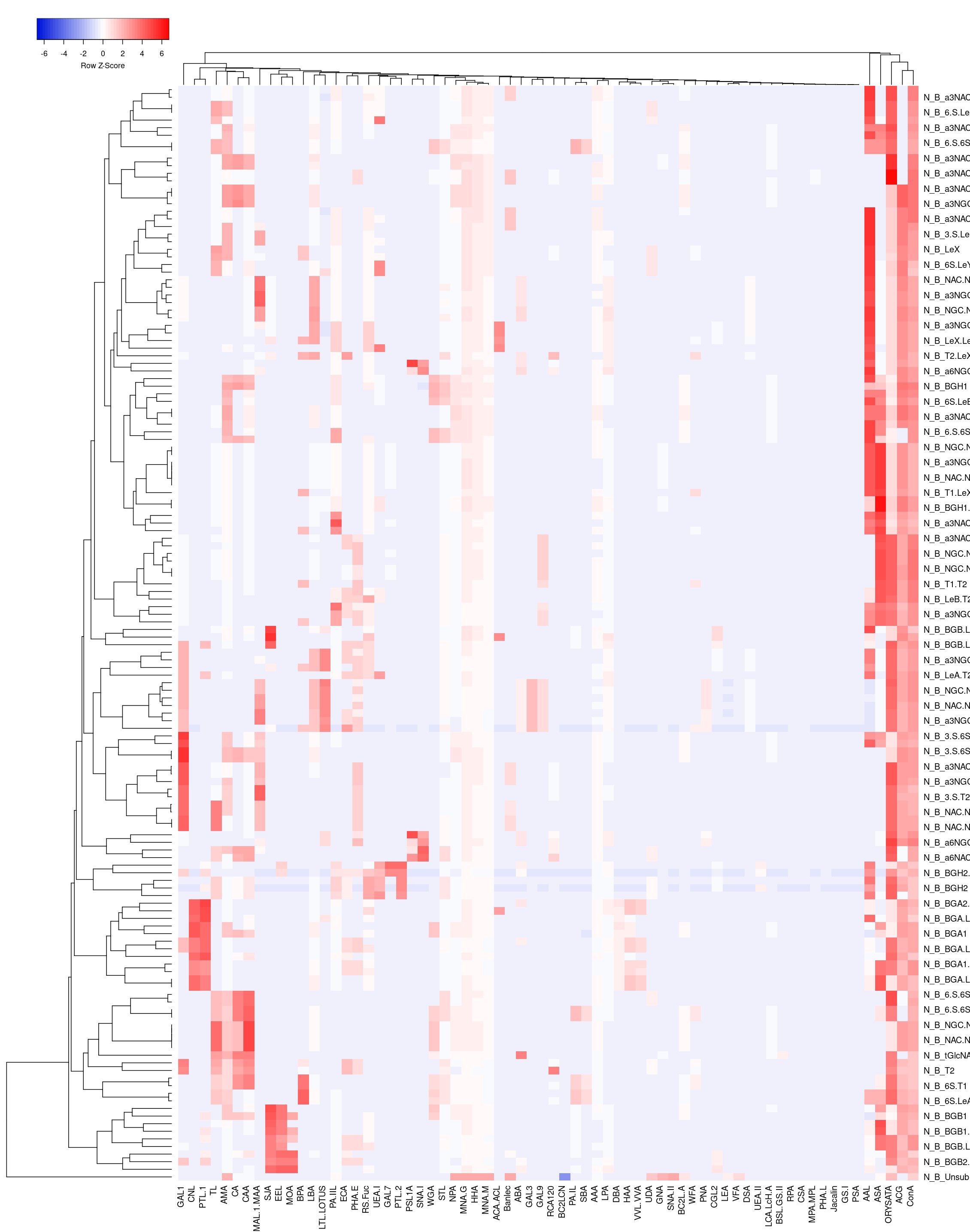


**(b)**


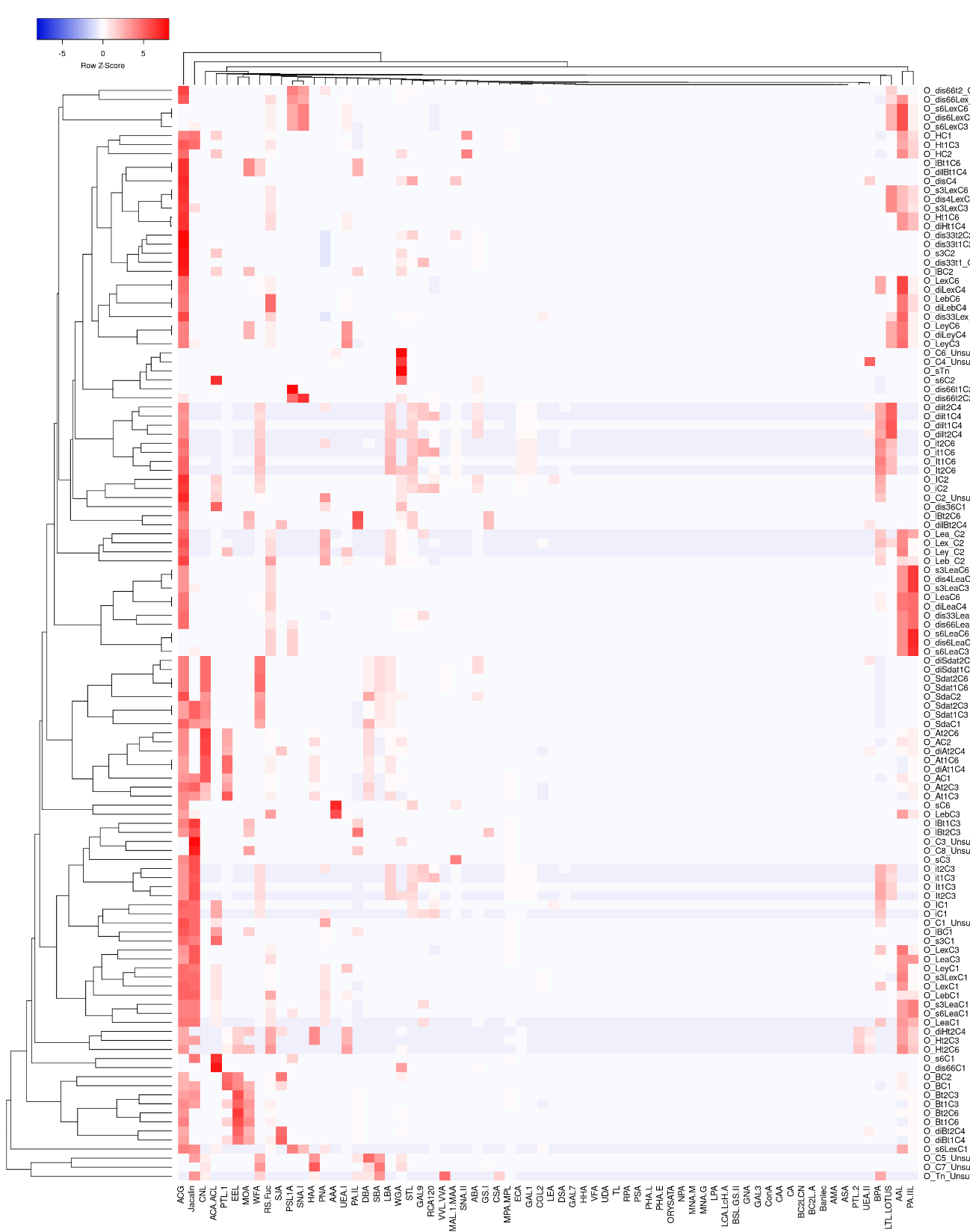

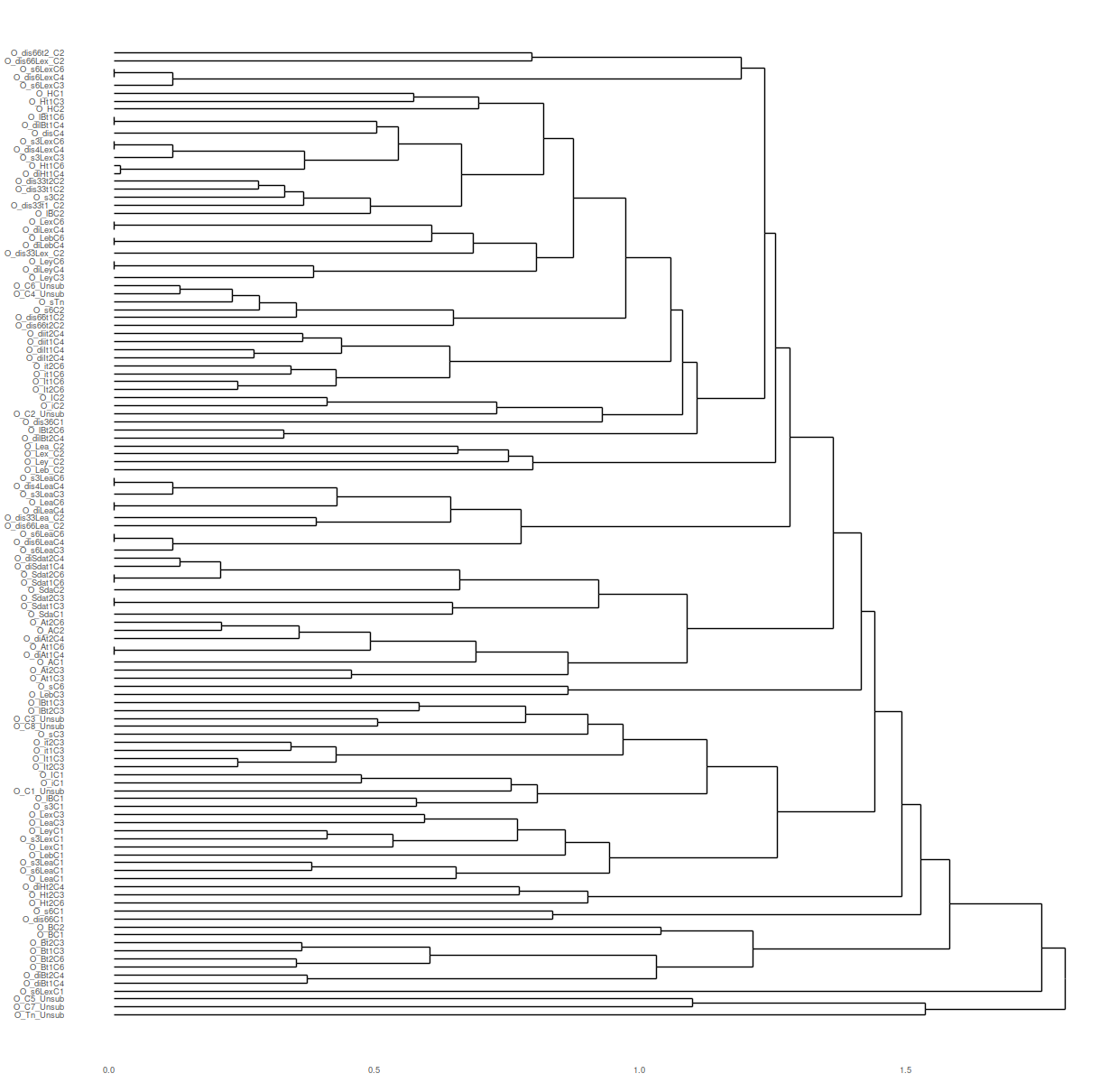

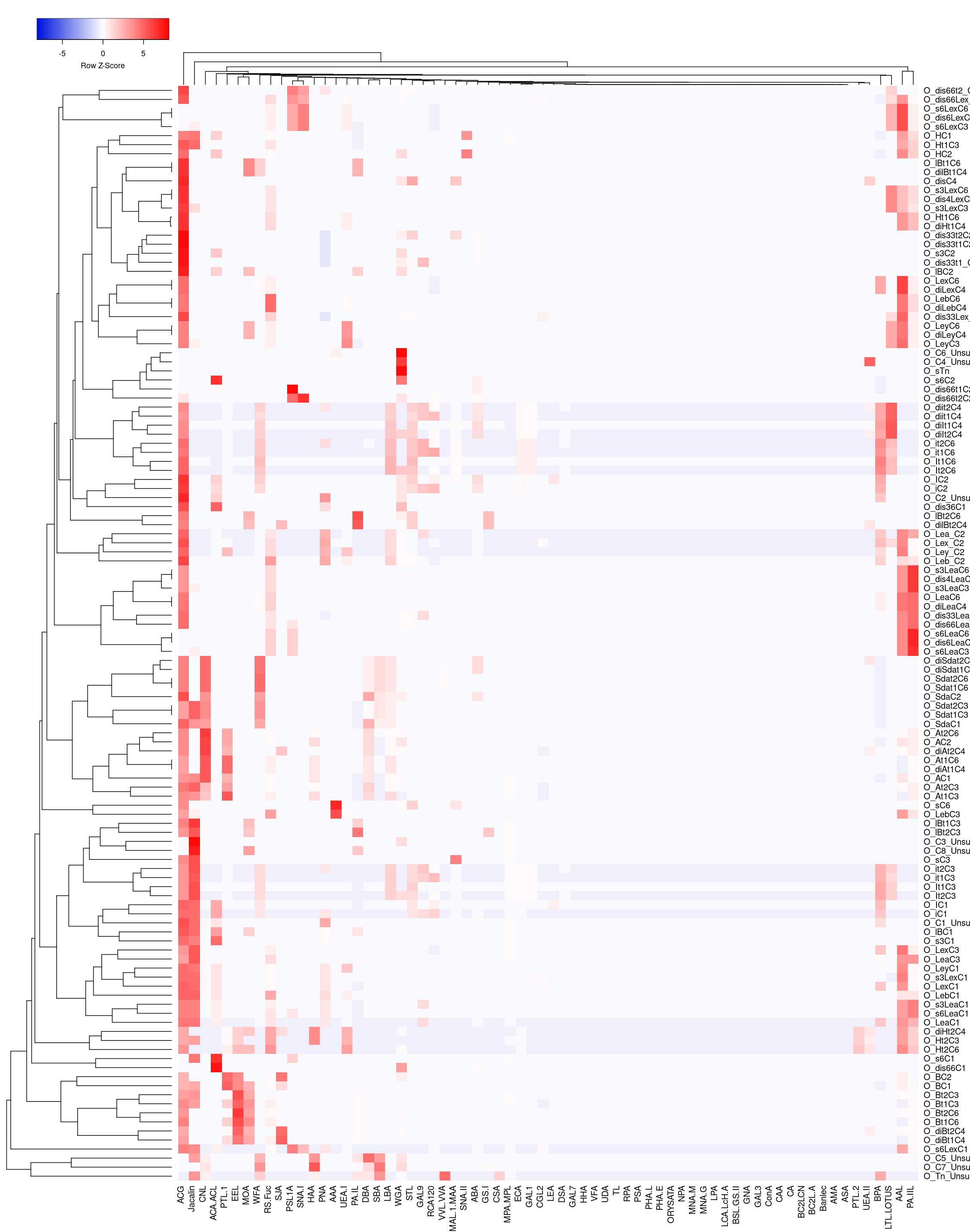

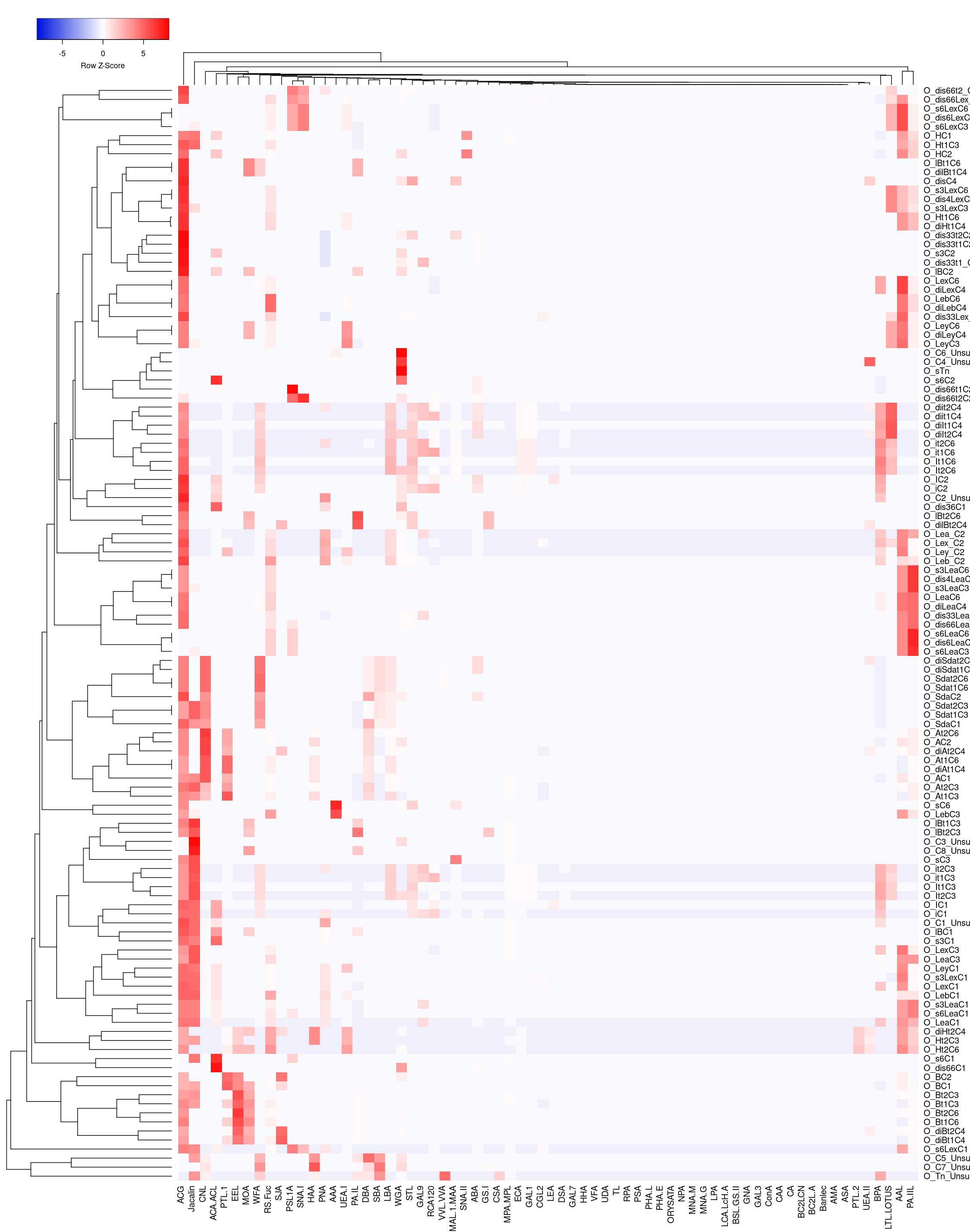


**Figure S1: Predicted binding of lectins to various glycans: (a)** A heat map showing the predicted binding propensities of 68 lectins (portrayed on the columns) to different *in silico*-generated N-extension sequences (portrayed on the rows). The Z-score is a measure of the number of standard deviations between an individual data value and the mean, and is given by the formula, Z = (X-µ)/σ, where X is the individual data value, µ is the population mean, and σ is the population standard deviation. **(b)** A heat map showing the predicted binding propensities of 68 lectins (portrayed on the columns) to different *in silico*-generated O-extension sequences (portrayed on the rows). The Z-score is a measure of the number of standard deviations between an individual data value and the mean, and is given by the formula, Z = (X-µ)/σ, where X is the individual data value, µ is the population mean, and σ is the population standard deviation.

**Figure S2**

**
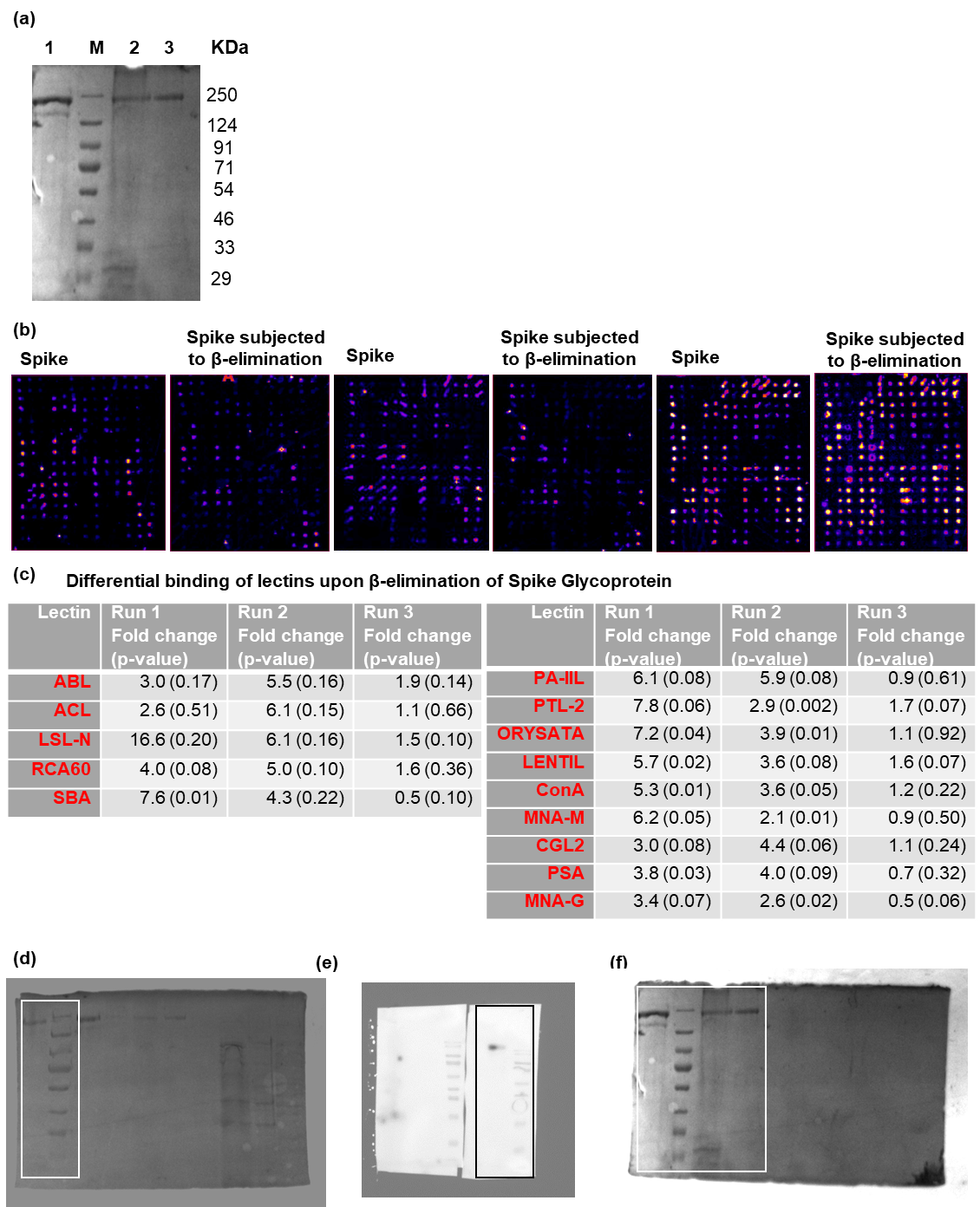
Figure S2:** **Binding of lectins to Spike glycoprotein subjected to β-elimination.** **(a)** SDS-PAGE of Spike glycoprotein (~140 KDa) expressed and purified from HEK293F cell (lane 1) and purified Spike glycoprotein after β-elimination to remove O-glycans (lanes 2 & 3). **b)** Pseudo-coloured images showing the binding of Spike glycoprotein with and without β-elimination (10 µg). The images were generated using the Protein Array Analyzer macro plugin in Image J. **(c)** Fold change and p-values observed for O-glycan binding lectins (left table) and other lectins (right table) in a paired t-test (n=3). (**d**) Uncropped image of Figure 4a with the cropped region shown in white outline. (**e**) Uncropped image of Figure 4b with the cropped region shown in black outline. (**f**) Uncropped image of Figure S2a with the cropped region shown in white outline. **Figure S3**

**
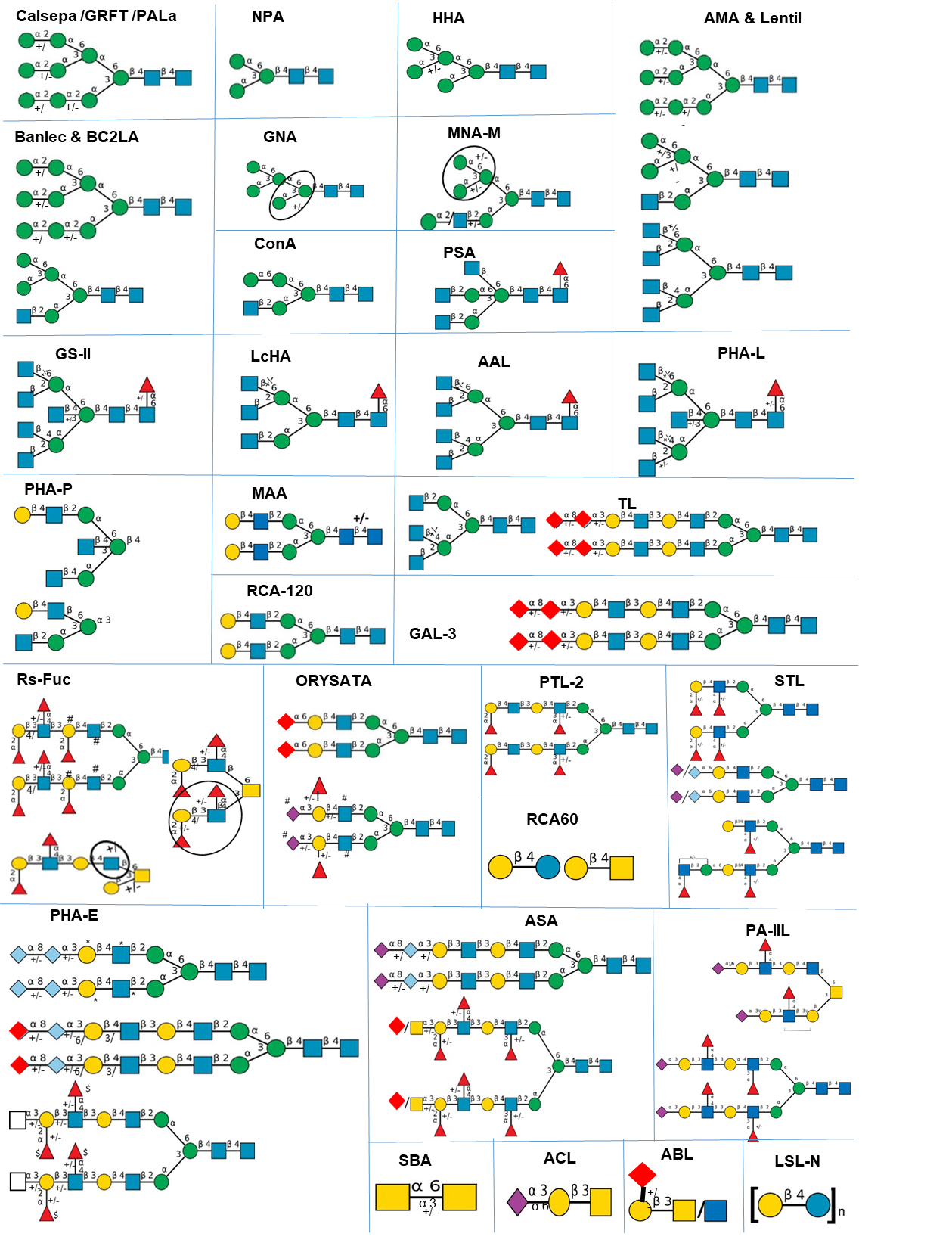
**

**Figure S3: Graphical representation of the top glycans predicted to be bound by each of the lectins that bind to SARS-CoV-2 Spike glycoprotein and/or cultured SARS-CoV-2 virus.** The list of all glycans evaluated using the MotifFinder software for prediction of binding by the lectins is available in **Supplementary Data 1 of Figures 2 and 3**. All MotifFinder models are available in **Supplementary Data 2**. The glycan motifs of all lectins evaluated in this study are provided in **Table S2**. Glycan structures were drawn as per SNFG notation using SNFG: Draw Glycan ^1^.

**Figure S4**

**
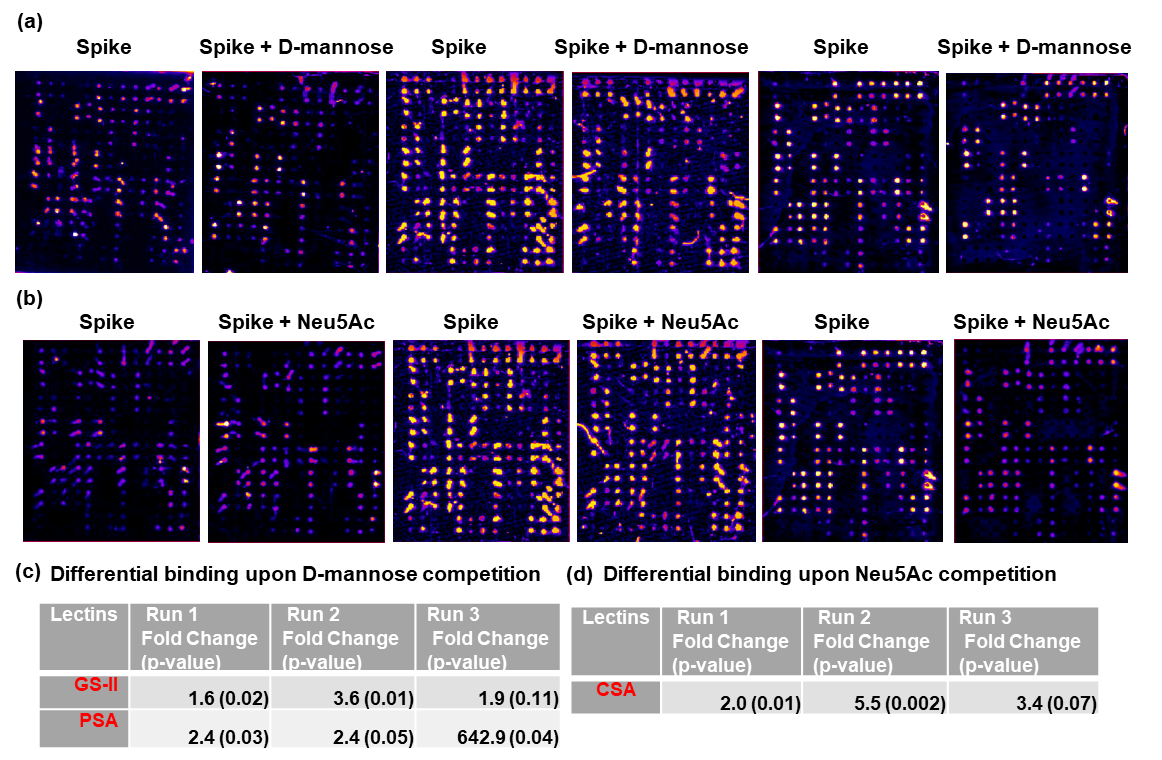
**

**Figure S4: Binding of lectins to Spike glycoprotein with D-mannose and Neu5Ac competition. (a)** Pseudo-coloured images showing the binding of Spike glycoprotein (10 µg) in the presence or absence of 500 mM mannose. **(b)** Pseudo-coloured images showing the binding of Spike glycoprotein (10 µg) in the presence or absence of 100 mM sialic acid. The images were generated using the Protein Array Analyzer macro plugin in Image J. **(c)** Fold change and p-values observed upon D-mannose competition in a paired t-test (n=3). **(d)** Fold change and p-values observed upon Neu5Ac competition in a paired t-test (n=3).

**Figure S5**

**
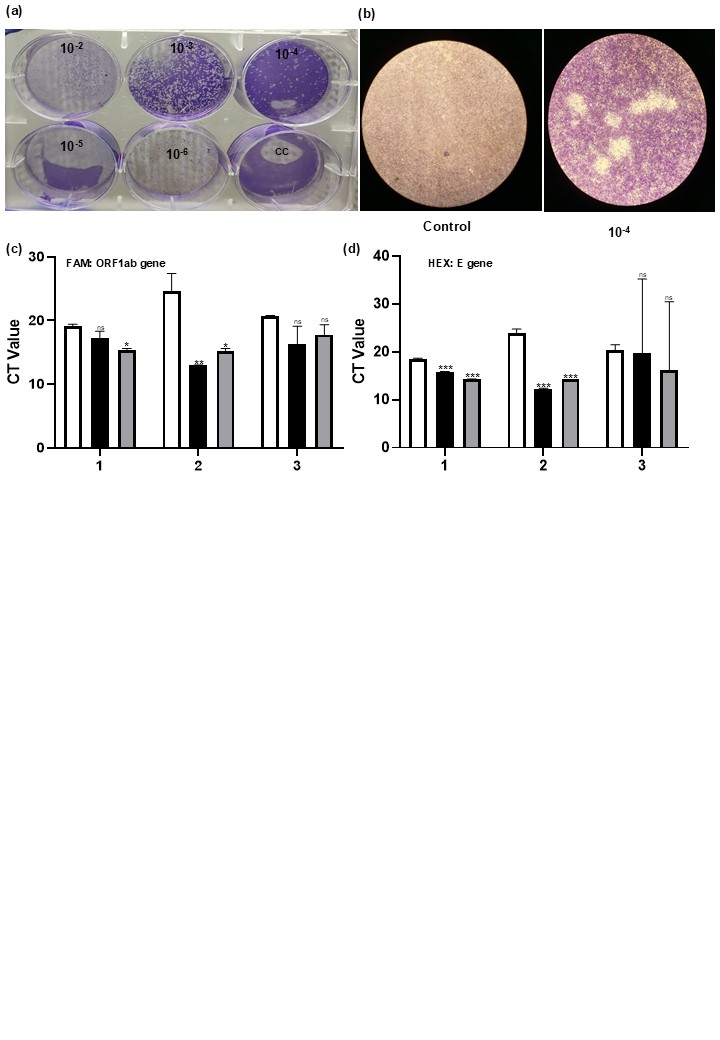
**

**Figure S5**: **Propagation and quantitation of enriched SARS-CoV-2.** **(a)** Quantification of SARS-CoV-2 virus titer propagated in Vero E6 cells. The plaque assay was performed with different dilutions of virus (ranging from 10^-2^ to 10^-6^) along with uninfected Vero E6 control (CC) in a 6-well plate (72 plaques were observed in dilution 10^-4^, hence the titer was calculated to be 1.8x10^6^ PFU/ml) **(b)** Plaques observed at 10X magnification (uninfected Vero E6 control and virus plated at 10^-4^ dilution) **(c,d)** RT-PCR to verify enrichment of virus in three biological replicates (referred to as 1, 2, and 3 on the x-axis). Un-enriched virus is indicated by white bars, PEG-enriched virus is indicated by black bars and Dynabead kit-enriched virus is indicated by grey bars. The RT-PCR Ct values were calculated for FAM representing the ORF1ab gene of SARS-CoV-2 **(c)** as well as for HEX representing the E gene of SARS Virus **(d)**, and the RT-PCR was performed with three technical replicates for each biological replicate. Error bars show standard deviation. “ns” indicates non-significant (P> 0.05), * indicates P≤ 0.05, ** indicates P ≤ 0.01, and *** indicates P ≤ 0.003.

**Figure S6**

**(a)**


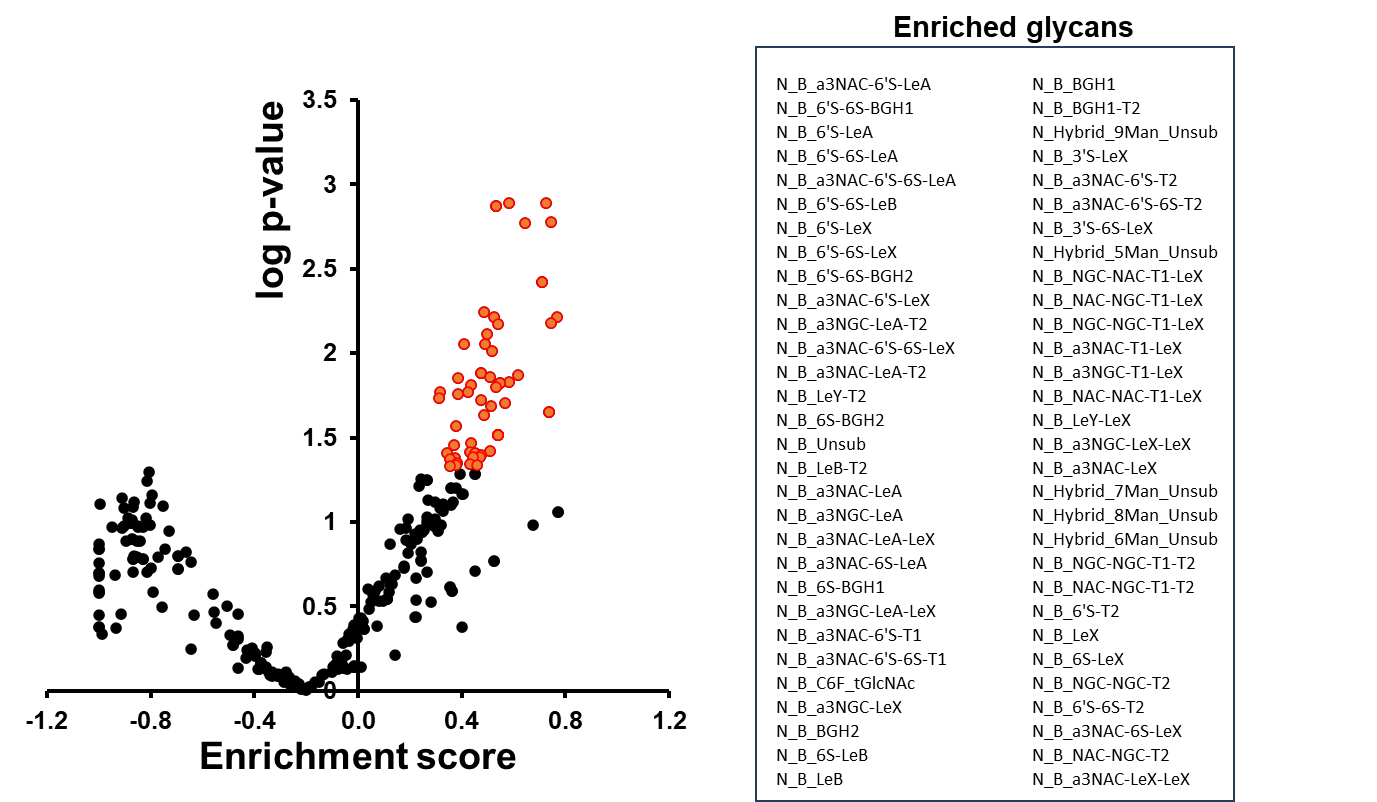


**(b)**


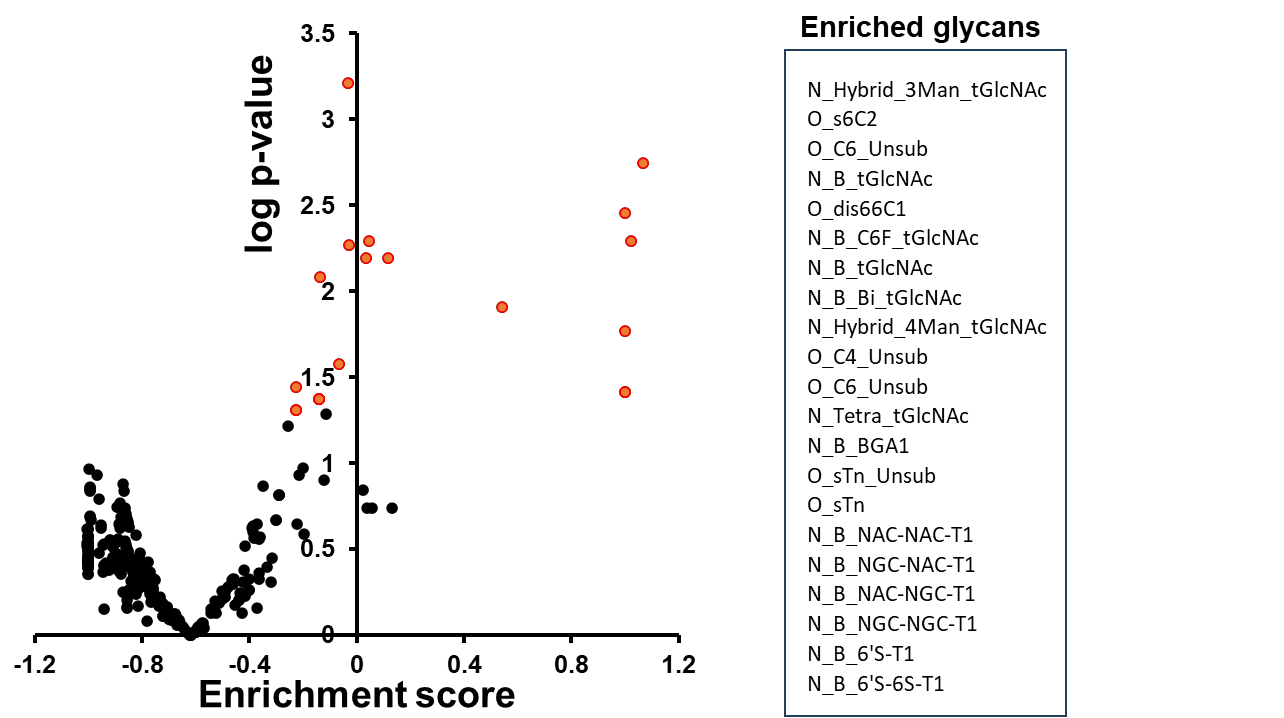


**Figure S6: Glycan motifs on SARS-CoV-2 Spike glycoprotein. (a)** Volcano plot analysis indicating the glycan motifs (in red; also listed to the right of the plot) found to be significantly enriched in the set of lectins listed in **Table S3** that bind with high intensity to SARS-CoV-2 Spike glycoprotein or SARS-CoV-2 virions (p-value <0.05, unpaired t-test with the remaining lectins). The enrichment score was obtained by calculating the difference in binding intensities observed for the top binding lectins (listed in **Table S3**) and the remaining lectins and dividing this by the total binding intensity observed for all lectins. **(b)** Volcano plot analysis indicating the glycan motifs (in red; also listed to the right of the plot) found to be significantly enriched in the set of lectins listed in **Table S4** that bind differentially to SARS-CoV-2 Spike glycoprotein and SARS-CoV-2 virions (p-value <0.05, unpaired t-test with the remaining lectins). The enrichment score was obtained by calculating the difference in binding intensities observed for the lectins that bound differentially to SARS-CoV-2 Spike glycoprotein and the SARS-CoV-2 virions (listed in **Table S4**) and the remaining lectins and dividing this by the total binding intensity observed for all lectins.

**Figure S7**

**
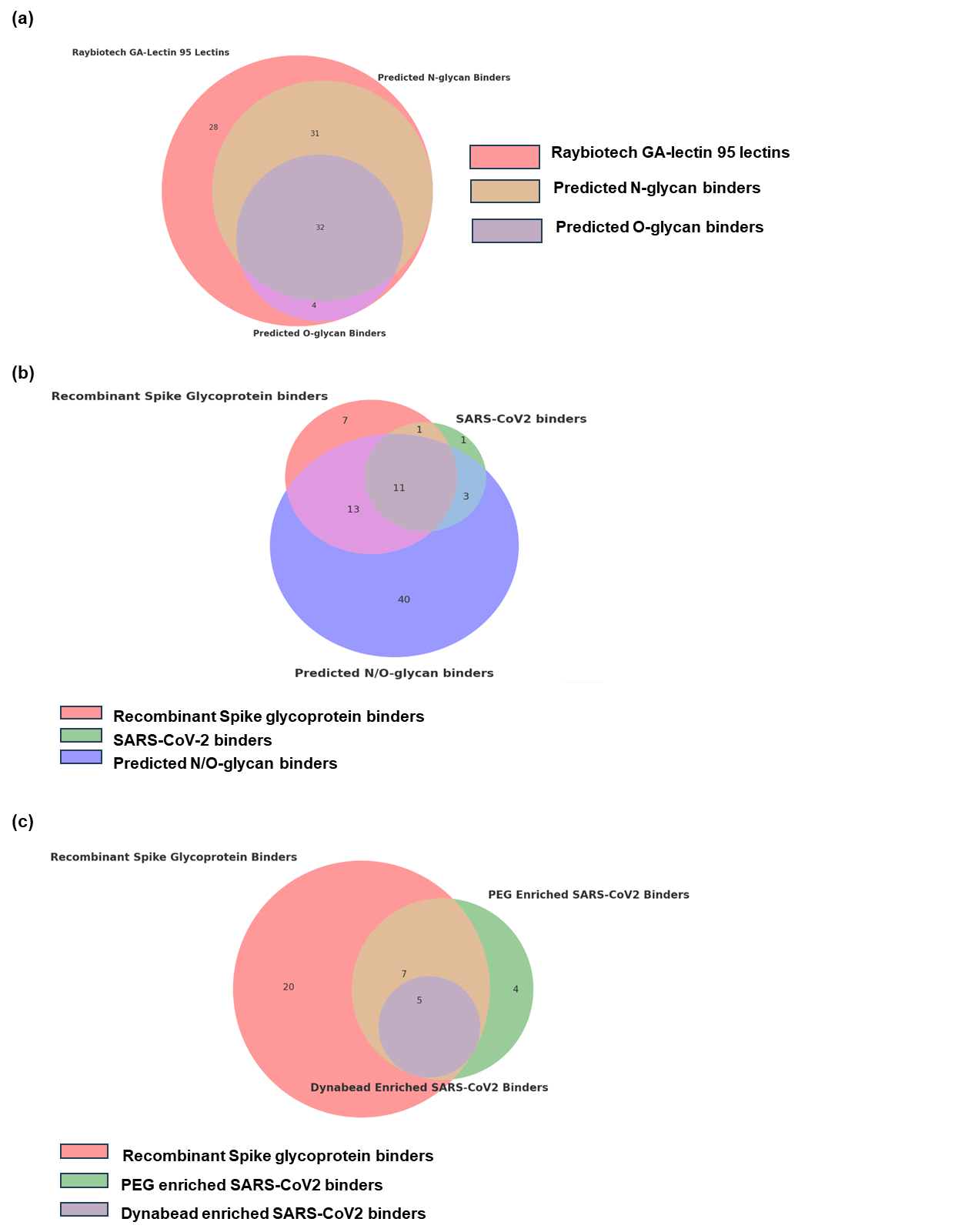
**

**Figure S7: Venn Diagrams showing how the results of the prediction of the glycan profiles of the lectins and the lectin array results inter-relate and complement with each other. (a)** Venn Diagram for the predicted N-glycan binders (both N-core and N-extension motifs; identified by MotifFinder analysis of publically available glycan array data), predicted O-glycan binders (both O-core and O-extension motifs; identified by MotifFinder analysis of publically available glycan array data), and the whole set of Raybiotech GA-lectin 95 lectins. **(b)** Venn Diagram for the predicted N/O-glycan binders, recombinant SARS-CoV-2 Spike glycoprotein binders (identified from the lectin array assays) and SARS-CoV2 virion binders (identified from the lectin array assays). **(c)** Venn Diagram for the recombinant SARS-CoV-2 Spike glycoprotein binders (identified from the lectin array assays), Dynabead kit-enriched SARS-CoV2 binders (identified from the lectin array assays) and PEG-enriched SARS-CoV2 binders (identified from the lectin array assays).

**Table S1: Lectins predicted to bind to *in silico* generated core N-glycans, N-glycan extension sequences, O-core glycans, and O-glycan extension sequences.**

| **Lectins** | **N-core** | **O-Core** | **N-Ext** | **O-Ext** |
| --- | --- | --- | --- | --- |
| **AAA** | **No** | **Yes** | **No** | **Yes** |
| **AAL** | **Yes** | **No** | **Yes** | **Yes** |
| **ABA** | **Yes** | **No** | **Yes** | **Yes** |
| **ACA/ACL** | **No** | **Yes** | **Yes** | **Yes** |
| **ACG** | **No** | **Yes** | **Yes** | **Yes** |
| **AMA** | **Yes** | **No** | **Yes** | **No** |
| **ASA** | **Yes** | **No** | **Yes** | **No** |
| **Banlec** | **Yes** | **No** | **Yes** | **No** |
| **BC2L-A** | **Yes** | **No** | **Yes** | **No** |
| **BC2LCN** | **Yes** | **No** | **No** | **No** |
| **BPA** | **No** | **Yes** | **Yes** | **Yes** |
| **BSL/GS-II** | **Yes** | **No** | **No** | **No** |
| **CA** | **Yes** | **No** | **Yes** | **No** |
| **CAA** | **Yes** | **No** | **Yes** | **No** |
| **CGL2** | **No** | **No** | **Yes** | **No** |
| **CNL** | **No** | **Yes** | **Yes** | **Yes** |
| **ConA** | **Yes** | **No** | **Yes** | **No** |
| **CSA** | **No** | **Yes** | **No** | **No** |
| **DBA** | **No** | **Yes** | **Yes** | **Yes** |
| **DSA** | **Yes** | **No** | **No** | **No** |
| **ECA** | **No** | **No** | **Yes** | **No** |
| **EEL** | **No** | **No** | **Yes** | **No** |
| **GAL1** | **No** | **No** | **Yes** | **No** |
| **GAL3** | **No** | **No** | **Yes** | **No** |
| **GAL7** | **No** | **No** | **Yes** | **No** |
| **GAL9** | **No** | **No** | **Yes** | **Yes** |
| **GNA** | **Yes** | **No** | **No** | **No** |
| **GS-I** | **No** | **No** | **No** | **Yes** |
| **HAA** | **No** | **Yes** | **Yes** | **Yes** |
| **HHA** | **Yes** | **No** | **Yes** | **No** |
| **Jacalin** | **No** | **Yes** | **No** | **Yes** |
| **LBA** | **Yes** | **No** | **Yes** | **Yes** |
| **LCA** | **Yes** | **No** | **No** | **No** |
| **LEA** | **Yes** | **No** | **No** | **Yes** |
| **LPA** | **No** | **No** | **Yes** | **No** |
| **LTL** | **Yes** | **No** | **Yes** | **Yes** |
| **MAL-1/MAA** | **No** | **No** | **Yes** | **Yes** |
| **MNA-G** | **Yes** | **No** | **Yes** | **No** |
| **MNA-M** | **Yes** | **No** | **Yes** | **No** |
| **MOA** | **No** | **Yes** | **Yes** | **Yes** |
| **MPA/MPL** | **No** | **No** | **No** | **No** |
| **NPA** | **Yes** | **No** | **Yes** | **No** |
| **ORYSATA** | **Yes** | **No** | **Yes** | **No** |
| **PA-IIL** | **Yes** | **No** | **Yes** | **Yes** |
| **PA-IL** | **No** | **Yes** | **Yes** | **Yes** |
| **PHA-E** | **Yes** | **No** | **Yes** | **No** |
| **PHA-L** | **Yes** | **No** | **No** | **No** |
| **PNA** | **No** | **Yes** | **Yes** | **Yes** |
| **PSA** | **Yes** | **No** | **No** | **No** |
| **PSL1A** | **No** | **No** | **Yes** | **Yes** |
| **PTL-1** | **No** | **No** | **Yes** | **Yes** |
| **PTL-2** | **No** | **No** | **Yes** | **Yes** |
| **RCA120** | **No** | **No** | **Yes** | **Yes** |
| **RPA** | **Yes** | **No** | **No** | **No** |
| **RS-Fuc** | **No** | **No** | **Yes** | **Yes** |
| **SBA** | **No** | **Yes** | **Yes** | **Yes** |
| **SJA** | **No** | **No** | **Yes** | **Yes** |
| **SNA-I** | **No** | **No** | **Yes** | **Yes** |
| **SNA-II** | **Yes** | **No** | **No** | **Yes** |
| **STL** | **No** | **No** | **Yes** | **Yes** |
| **TL** | **Yes** | **No** | **Yes** | **No** |
| **UDA** | **Yes** | **No** | **Yes** | **No** |
| **UEA I** | **No** | **No** | **Yes** | **Yes** |
| **UEA-II** | **No** | **Yes** | **Yes** | **Yes** |
| **VFA** | **Yes** | **No** | **No** | **No** |
| **VVL/VVA** | **No** | **Yes** | **Yes** | **No** |
| **WFA** | **No** | **Yes** | **Yes** | **Yes** |
| **WGA** | **Yes** | **Yes** | **Yes** | **Yes** |

**Table S2: Carbohydrate-binding specificities of the lectins on the array used in this study.** The carbohydrate-binding specificities are provided as per the Ray Biotech lectin array manual and as per the Motif Finder analysis conducted in this study (listing all glycan motifs predicted with a binding intensity >0.33 with a p-value <0.05 in each lectin model by the MotifFinder algorithm).

| **Lectin** | **Carbohydrate specificity as per Ray Biotech Lectin Array Manual** | **Predicted carbohydrate specificity following MotifFinder analysis in this study (Relative binding >0.33 with p-value <0.05 in each lectin model generated using MotifFinder)** |
| --- | --- | --- |
| AAA | αFuc | Terminal β-GlcNAc Non-N-Glycan |
| AAL | Fucα6GlcNAc | N-glycan α6 core fucose, Bisecting or triantennary 6' N-glycan α6 core fucose, Lewis A, terminal Lewis A, terminal Lewis B, terminal or terminal O-GlcNAc core or internal or 3' sulfo or sialyl Lewis X, terminal Lewis Y, Blood group H type 2 or type 1 or O-GlcNAc core or O-GalNAc core 1, Blood group A, Blood group B, Lacto glycosphingolipid |
| ABA | Gal-β-1,3-GalNAc | Terminal N-Glycan GlcNAc, Terminal N-Glycan α-GlcNAc |
| ABL | Gal-β-1,3-GalNAc, Gal-β1,3-GlcNAc | Not analyzed by MotifFinder |
| ACG | α2-3 Sialic Acid | Final Motif report could not be generated using MotifFinder |
| ACL | Galβ3GalNAc | α3 Sialyl galactose O-GalNAc core 1, O-GalNAc sialyl GalNAc core 1, O-GalNAc core 1, internal Lewis x |
| AMA | Mannose | N-glycan mannose A2 dimer, terminal mannosyl core, N-glycan terminal GlcNAc, N-glycan terminal β-Gal |
| ASA | αMan | Type 1 - Type 2 DiLacNAc, Lewis A |
| BANLEC | containing α1,3-glycoside bond | Terminal mannose, O-mannose, bisecting N-glycan |
| BC2L-A | High-mannose | High mannosyl core, terminal mannose |
| BC2LCN | Fucα1-2Galβ1-3GalNAc (H type 3), Fucα1-2Galβ1-3GlcNAc (H type 1) | Final Motif report could not be generated using MotifFinder |
| BPA | Galβ3GalNAc | Terminal GalNAc, terminal type 1 LacNAc, terminal Lewis x, terminal Galβ1-3/4N-Acetylhexosamine |
| CA | Lac > GalNAc > Gal | Terminal N-Glycan GlcNAc, biantennary N-glycan terminal type 1 LacNAc, terminal type 2 LacNAc biantennary N-glycan, α6 sialyl type 2 LacNAc, N-glycan Lewis A, triantennary 3' N-glycan, (biantennary) N-glycan α6 core fucose |
| CAA | GalNAc | Terminal N-glycan GlcNAc, terminal type 1 LacNAc, biantennary N-glycan α-fuc, terminal type 2 LacNAc biantennary N-glycan, α6 sialyl type 2 LacNAc, triantennary 3' N-glycan |
| CALSEPA | High-mannose | Not analyzed by MotifFinder |
| CGL2 | βGal, GalNAcα1-3Gal (Blood Group A), Galα1-3Gal (Blood Group B) | α-fuc terminal GalNAc |
| CNL | 2]Galβ1-4GlcNAc (Blood Group A) | Terminal Type 2 LacDiNAc, Terminal GalNAc, Terminal GalNAc O-GalNAc, Terminal GalNAc 5-Acetyl Neuraminic Acid, Blood Group A, 6’ Sulfated Gal or GalNAc |
| Con A | αMan, αGlc | Terminal mannose N-glycan, terminal mannosyl core, mannose a2 dimer, terminal GlcNAc N-glycan, biantennary N-glycan with terminal β-GlcNAc, hybrid or complex N-glycan with terminal sialyl type 2 LacNAc or sialyl Lewis X type 2 LacNAc or Lewis X, N-glycan with α6 core fucose, type 2 DiLacNAc |
| CPA | Fetuin | Not analyzed by MotifFinder |
| CSA | GalNAc | Terminal type 2 LacDiNAc, terminal GalNAc |
| DBA | αGalNAc | TerminalGalNAc, terminal GalNAc a-series ganglioside |
| DISCOIDIN I | αGalNAc (Tn antigen), LacNAc | Not analyzed by MotifFinder |
| DISCOIDIN II | Gal, LacNAc, Asialoglycans, Gal/GalNAcβ1-4GlcNAcβ1-6Gal/GalNAc | Not analyzed by MotifFinder |
| DSA | (GlcNAc)2-4 | Tetraantennary N-glycan, terminal N-glycan GlcNAc, triantennary 6' N-glycan |
| ECA | Galβ4GlcNAc | Type 2 DiLacNAc, Tetraantennary N-Glycan, Terminal Type 2 LacNAc, Terminal Type 2 LacNAc Biantennary N-Glycan, Terminal Type 2 LacDiNAc |
| EEL | Galα3Gal | Blood group B, blood group B type 1, blood group H type 2 |
| F17AG | GlcNAc | Not analyzed by MotifFinder |
| GAL1 | branched LacNAc, Gal | Blood Group H O-GlcNAc Core, O-GalNAc Core 2, Blood Group H Type 2 DiLacNAc |
| GAL1-S | branched LacNAc | Not analyzed by MotifFinder |
| GAL2 | GalNAcα1-3Gal (Blood Group A), branched LacNAc | Not analyzed by MotifFinder |
| GAL3 | poly LacNAc | Type 2 DiLacNAc terminal type 1 LacNAc, type 2 DiLacNAc terminal type 2 LacNAc, type 2 TriLacNAc/i antigen, Blood Group H Type 2 α6 Sialyl Type 2 LacNAc, Neolacto Glycosphingolipid Terminal Type 2 LacNAc |
| GAL3C-S | poly LacNAc | Not analyzed by MotifFinder |
| GAL7-S | Galβ1-3GlcNAc | Blood Group H Type 2 |
| GAL9 | poly LacNAc, GalNAcα1-3Gal (Blood Group A) | Blood Group H Type 2 Type 2 TriLacNAc/i Antigen |
| GHA | Gal, methyl α-D-galactopyranoside, GalNAc | Not analyzed by MotifFinder |
| GNA | αMan | High Mannosyl Core, Terminal Mannosyl Core, Terminal Mannose and 5-Acetyl Neuraminic Acid |
| GRFT | high mannose | Not analyzed by MotifFinder |
| GS-I | αGal, α3GalNAc | Tn Antigen, Terminal α-Gal, O-GlcNAc Core Blood Group H Type 2, Terminal α-Gal O-GlcNAc Core, Blood Group A, Terminal GalNAc |
| GS-II or BSL2 | α or βGlcNAc | Type 2 TriLacNAc/I antigen, terminal β-GlcNAc type 2 TriLacNAc/i antigen, terminal β-GlcNAc type 2 DiLacNAc, terminal N-glycan GlcNAc, terminal α/β-GlcNAc non-N-glycan |
| HAA | GalNAc | No motifs with low p-values |
| HHA | αMan | N-glycan terminal mannosyl core, O-mannose, O-mannose high mannosyl core, mannose a2 dimer N-glycan, terminal mannose O-mannose, chitose non-N-glycan, terminal mannose chitose |
| HMA | Neu5Ac, GalNAc | Not analyzed by MotifFinder |
| IRA | GalNAc | Not analyzed by MotifFinder |
| Jacalin | Galβ3GalNAc | O-GalNAc core 3, O-GalNAc core 1, O-GalNAc terminal GalNAc, O-GalNAc core 3 2'-fucosylated, O-GalNAc core 1 2'-fucosylated |
| LAL | α-Me-L-Fucose among monosaccharides | Not analyzed by MotifFinder |
| LBA | GalNAcα(1,3)[αFuc(1,2]Gal | Terminal N-Glycan GlcNAc, Type 2 DiLacNAc Biantennary N-Glycan |
| LcH A | αMan, αGlc | N-Glycan α6 Core Fucose |
| LEA | (GlcNAc)2-4 | Terminal β-GlcNAc Non-N-Glycan, α3 Sialyl Type 2 LacNAc |
| LENTIL | D-Man, D-Glc | Not analyzed by MotifFinder |
| Lotus | αFuc | Terminal β-GlcNAc Non-N-Glycan |
| LPA | Neu5Ac | Terminal α-GlcNAc, Type 2 TriLacNAc/i Antigen |
| LSL-N | LacNAc, poly LacNAc | Not analyzed by MotifFinder |
| MAA | Galβ4GlcNAc | α3 Sialyl Type 2 LacNAc, α3 Sialyl Type 2 LacNAc O-GlcNAc Core, PolySialyl Galactose, Terminal 3’ Sulfated Galactose, 3’ Sulfo Lewis X, Neolacto Glycosphingolipid |
| MALECTIN | Glc2-N-biose | Not analyzed by MotifFinder |
| MNA-G | Gal | High mannosyl core |
| MNA-M | Man | High mannosyl core |
| MOA | 3Galβ1-4GlcNAc, Galα1-3Gal | Blood Group B Type 2, Blood Group B, Blood group H type 2 |
| MPL | Galβ3GalNAc | O-GalNAc Terminal N-Acetyl Hexosamine, O-GalNAc Core 1, O-GalNAc Core 3 |
| NPA | αMan | N-glycan terminal mannosyl core, high mannosyl core |
| ORYSATA | High-mannose | Biantennary N-Glycan α6 Sialyl Type 2 LacNAc, N-Glycan Terminal Type 1 LacNAc, Biantennary N-Glycan, Chitose Terminal Mannose |
| PA-IIL | Fuc, fucose containing oligosaccharides | Sialyl Lewis A, Lewis A, α-Fuc 6’ Sulfated Gal or GalNAc, Blood Group H Type 2 |
| PA-IL | Galα1-3(4)Gal | Terminal α-Gal O-GlcNAc Core, Terminal α-Gal, Biantennary N-Glycan Terminal Type 1 LacNAc |
| PALa | High-mannose | Not analyzed by MotifFinder |
| PHA-E | Galβ4GlcNAcβ2Manα6(GlcNAcβ4) (GlcNAcβ4Manα3)Manβ4 | Bisecting N-Glycan, Biantennary N-Glycan Terminal Hexose |
| PHA-L | Galβ4GlcNAcβ6(Gl cNAcβ2Manα3)Manα3 | Triantennary 6’ N-Glycan, Bisecting N-Glycan, Triantennary 6’ N-Glycan, Tetraantennary N-Glycan |
| PHA-P | Galβ4GlcNAcβ2Manα6(GlcNAcβ4) (GlcNAcβ4Manα3)Manβ4, Galβ4GlcNAcβ6(GlcNAcβ2Manα3)Manα3 | Not analyzed by MotifFinder |
| PNA | Galβ3GalNAc | No motifs with low p-values |
| PPL | α/βGalNAc | Not analyzed by MotifFinder |
| PSA | αMan, αGlc | N-Glycan α6 Core Fucose, Triantennary 6’ N-Glycan |
| PSL1A | α2-6 Sialic Acid | α6 Sialyl Type 2 LacNAc Type 2 DiLacNAc, α6 Sialyl Type 2 LacNAc Type 2 TriLacNAc/i Antigen, α6 Sialyl Type 2 LacNAc |
| PTL-1 | GalNAc, Gal | Blood group A, Blood group A type 1, Blood Group A type 2, Blood group B, type 2 DiLacNAc neolacto glycosphingolipid |
| PTL-2 | Blood group H structures and the T-antigen | Blood group H type 2 |
| PWA | GlcNAc(β1,4) GlcNAc oligomers, [Gal-(β1,4) | Not analyzed by MotifFinder |
| RCA 120 | Gal, Lac | Terminal Type 2 LacNAc Biantennary N-Glycan, Terminal Type 2 LacNAc O-GalNAc Core 2, Terminal Type 2 LacNAc N-Glycan α6 Core Fucose, Terminal Type 2 LacNAc Triantennary 3’ N-Glycan, Terminal Type 2 LacNAc α-Fuc, α6 Sialyl Type 2 LacNAc |
| RCA 60 | Gal, GalNAc, lactose | Not analyzed by MotifFinder |
| RPA | GalNAc, thyroglobulin | Tetraantennary N-Glycan |
| RS-FUC | Fuc | α-fuc, O-GlcNAc Core Blood Group H, blood group H, Lewis X, GlcNAc Base Terminal Type 1 LacNAc |
| SAMB | NeuAcα2-6Gal/GalNAc | Not analyzed by MotifFinder |
| SBA | α > β GalNAc | Terminal Type 2 LacDiNAc |
| SHA | GalNAc | Not analyzed by MotifFinder |
| SJA | β-GalNAc | Final Motif report could not be generated using MotifFinder |
| SNA-I | NANA α(2,6)GalNAc > GalNAc = Lac > | N-Glycan α6 Core Fucose, α6 Sialyl Type 2 LacNAc Type 2 TriLacNAc/i Antigen, α6 Sialyl Type 2 LacNAc, α6 Sialyl Type 2 LacNAc O-GalNAc, α6 Sialyl Galactose O-GlcNAc Core, α6 Sialyl Type 2 LacNAc, α6 Sialyl Type 2 LacNAc Lacto Glycosphingolipid |
| SNA-II | GalNAc > Gal | Terminal Mannosyl Core, mannose a2 dimer, terminal mannose, blood group H a-series ganglioside, blood group H O-GalNAc core 1 |
| SSA | GalNAc | Not analyzed by MotifFinder |
| STL | (GlcNAc)2-4 | Type 2 DiLacNAc O-GlcNAc Core, O-GlcNAc Core Neu5Ac, O-GlcNAc Core Terminal β-Gal, GlcNAc Base Terminal Type 2 LacDiNAc, Terminal α-Gal O-GlcNAc Core, Terminal β GlcNAc Non-N-Glycan, Type 2 TriLacNAc/i Antigen |
| TL | GalNAc | N-Glycan α6 Core Fucose, type 2 TriLacNAc/I antigen, Terminal N-Glycan GlcNAc, Terminal Type 2 LacNAc Biantennary N-Glycan, Biantennary N-Glycan Terminal β-Gal, Blood Group H N-Glycan, α6 Sialyl Type 2 LacNAc, Triantennary 6’ N-Glycan |
| UDA | GlcNAc | Terminal Mannose |
| UEA I | α-Fuc | Blood Group H Type 2, Neolacto Glycosphingolipid, Blood Group H Type 2 DiLacNAc, Blood Group H, Terminal Lewis Y, α-Fuc 6’ Sulfated Gal or GalNAc |
| UEA-II | Poly β(1,4)GlcNAc | Terminal β-GlcNAc Type 2 DiLacNAc, Terminal β-GlcNAc O-GalNAc Core 2,Terminal β-GlcNAc O-GlcNAc Core |
| VFA | α-Man | Terminal Man |
| VRA | α-Gal | Not analyzed by MotifFinder |
| VVA | GalNAc | Tn Antigen, Terminal Type 2 LacDiNAc, Terminal GalNAc |
| VVA-M | Man | Not analyzed by MotifFinder |
| WFA | GalNAc | Terminal Type 2 LacDiNAc, a-Series Ganglioside, Terminal GalNAc Neu5Ac, PolySialyl Gal, Type 2 TriLacNAc/i Antigen, Terminal Type 2 LacNAc N-Glycan, TerminalGalNAc, Tn Antigen |
| WGA | GlcNAc | Terminalβ-GalNAc Non-N-Glycan, Type 2 DiLacNAc Terminal beta β-GalNAc, Terminal β-GalNAcO-GlcNAc Core,Biantennary N-Glycan Terminalβ-GalNAc, Terminal Type 2 LacDiNAc, GlcNAc Base 6’ Sulfated Gal or GalNAc, Terminal α-GlcNAc, Monoantennary N-Glycan, Blood Group A Type 2, Terminal Type 2 LacNAc O-GlcNAc Core, α-Fuc Terminal GalNAc |

**Table S3: Lectins that bind with high intensity to SARS-CoV-2 Spike glycoprotein or SARS-CoV-2 virions, as identified in this study by lectin array analysis.** All lectins that bound significantly with respect to the negative control (p-value <0.05, unpaired t-test) and with average normalized percent relative fluorescence units (RFU) >100% to the Spike glycoprotein (Figure 4e) or to the PEG-enriched SARS-CoV-2 virions (Figure 7d) or to the Dynabead virus enrichment kit subjected SARS-CoV-2 virions (Figure 7d) were included in this list.

| **Lectins binding with high intensity to the Spike glycoprotein or SARS-CoV-2 virions** | |
| --- | --- |
| BC2L-A | LcH A |
| ABL | RCA 120 |
| ACL | ORYSATA |
| CALSEPA | Con A |
| PALa | AMA |
| GS-II | GNA |
| CA | TL |
| MNA-M | STL |
| PSA | AAL |
| LSL-N | ASA |
| LENTIL | SBA |
| GRFT | PA-IIL |
| GAL3 | NPA |
| PHA-L | CPA |
| HHA | PTL-2 |
| RS-FUC | PHA-P |
| PHA-E | MAA |
| RCA 60 | BANLEC |

**Table S4: Lectins that bind differentially to SARS-CoV-2 Spike glycoprotein and SARS-CoV-2 virions (as identified by a >5-fold change and p-value <0.05 in an unpaired t-test assuming homoscedasticity).**

| **Lectins** | **Fold change (Spike vs virus)** | **p-value (unpaired t-test assuming homoscedasticity; Spike vs virus)** |
| --- | --- | --- |
| AAA | 36.3 | 0.01 |
| ABA | 11.4 | 0.003 |
| ABL | 27.1 | 0.001 |
| ACL | 56.4 | 0.001 |
| BC2L-A | 8.7 | 0.01 |
| CA | 13.0 | 0.01 |
| CAA | 21.0 | 0.0004 |
| CALSEPA | 8.3 | 0.003 |
| CGL2 | 5.8 | 0.00005 |
| CNL | 7.7 | 0.003 |
| GRFT | 9.1 | 0.03 |
| GS-II | 24.2 | 0.005 |
| LENTIL | 5.2 | 0.03 |
| LSL-N | 17.1 | 0.02 |
| MALECTIN | 13.6 | 0.02 |
| MNA-G | 10.6 | 0.002 |
| MNA-M | 10.3 | 0.01 |
| MPL | 23.8 | 0.02 |
| PALa | 11.9 | 0.005 |
| PSA | 14.5 | 0.02 |
| WGA | 7.6 | 0.004 |

**Table S5: Glycans identified by mass spectrometry on SARS-CoV-2 Spike glycoprotein by previous studies ^2-6^**.

| **Unique glycans reported on Spike glycoprotein** | | | |
| --- | --- | --- | --- |
| **Afucosylated and asialylated** | **Fucosylated** | **Sialylated** | **Fucosylated and sialylated** |
| HexNAc(1) | HexNAc(2)Fuc(1)Hex(3)HexNAc(2) | HexNAc(1)Hex(1)NeuAc(1) | HexNAc(2)Fuc(1)Hex(3)HexNAc(2)Hex(2)NeuAc(1) |
| HexNAc(1)Hex(1) | HexNAc(2)Fuc(1)Hex(3)HexNAc(2)Hex(2) | HexNAc(3)Hex(4)NeuAc(1) | HexNAc(2)Fuc(1)Hex(3)HexNAc(4)Hex(4)NeuAc(4) |
| HexNAc(2) | HexNAc(2)Fuc(1)Hex(4)HexNAc(1)Hex(1) | HexNAc(3)Hex(5)NeuAc(1) | HexNAc(3)Hex(4)Fuc(1)NeuAc(1) |
| HexNAc(2)Hex(1) | HexNAc(2)Hex(3)Fuc(1) | HexNAc(3)Hex(6)NeuAc(1) | HexNAc(3)Hex(6)Fuc(1)NeuAc(1) |
| HexNAc(2)Hex(3) | HexNAc(3)Hex(3)Fuc(1) | HexNAc(3Hex(6)NeuAc(1) | HexNAc(4)Hex(4)Fuc(1)NeuAc(1) |
| HexNAc(2)Hex(3)HexNAc(1)Hex(1) | HexNAc(3)Hex(4)Fuc(1) | HexNAc(4)Hex(3)NeuAc(1) | HexNAc(4)Hex(4)Fuc(2)NeuAC(1) |
| HexNAc(2)Hex(3)HexNAc(3) | HexNAc(3)Hex(5)Fuc(1) | HexNAc(4)Hex(4)NeuAc(1) | HexNAc(4)Hex(5)Fuc(1)NeuAc(1) |
| HexNAc(2)Hex(4) | HexNAc(3)Hex(6)Fuc(1) | HexNAc(4)Hex(5)NeuAc(1) | HexNAc(4)Hex(5)Fuc(1)NeuAc(2) |
| HexNAc(2)Hex(5) | HexNAc(4)Hex(3)Fuc(1) | HexNAc(4)Hex(5)NeuAc(2) | HexNAc(5)Hex(3)Fuc(1)NeuAc(1) |
| HexNAc(2)Hex(6) | HexNAc(4)Hex(4)Fuc(1) | HexNAc(4)Hex(6)NeuAC(1) | HexNAc(5)Hex(4)Fuc(1)NeuAc(1) |
| HexNAc(2)Hex(7) | HexNAc(4)Hex(4)Fuc(2) | HexNAc(5)Hex(4)NeuAc(1) | HexNAc(5)Hex(5)Fuc(1)NeuAc(1) |
| HexNAc(2)Hex(8) | HexNAc(4)Hex(5)Fuc(1) | HexNAc(5)Hex(5)NeuAc(1) | HexNAc(5)Hex(5)Fuc(1)NeuAc(2) |
| HexNAc(2)Hex(9) | HexNAc(5)Hex(3)Fuc(1) | HexNAc(5)Hex(5)NeuAc(2) | HexNAc(5)Hex(6)Fuc(1)NeuAc(1) |
| HexNAc(2)Hex(10) | HexNAc(5)Hex(4)Fuc(1) | HexNAc(5)Hex(6)NeuAc(1) | HexNAc(5)Hex(6)Fuc(1)NeuAc(2) |
| HexNAc(3)Hex(3) | HexNAc(5)Hex(4)Fuc(2) | HexNAc(5)Hex(6)NeuAc(2) | HexNAc(5)Hex(6)Fuc(1)NeuAc(3) |
| HexNAc(3)Hex(4) | HexNAc(5)Hex(5)Fuc(1) | HexNAc(6)Hex(4)NeuAc(1) | HexNAc(6)Hex(5)Fuc(1)NeuAc(1) |
| HexNAc(3)Hex(5) | HexNAc(5)Hex(6)Fuc(1) | HexNAc(6)Hex(6)NeuAc(1) | HexNAc(6)Hex(5)Fuc(1)NeuAc(2) |
| HexNAc(3)Hex(6) | HexNAc(5)Hex(6)Fuc(1)Neu5Ac(1) | HexNAc(6)Hex(7)NeuAc(1) | HexNAc(6)Hex(5)Fuc(1)NeuAc(3) |
| HexNAc(4)Hex(3) | HexNAc(6)Hex(3)Fuc(1) |  | HexNAc(6)Hex(6)Fuc(1)NeuAc(1) |
| HexNAc(4)Hex(4) | HexNAc(6)Hex(4)Fuc(1) |  | HexNAc(6)Hex(6)Fuc(1)NeuAc(2) |
| HexNAc(4)Hex(5) | HexNAc(6)Hex(5)Fuc(1) |  | HexNAc(6)Hex(6)Fuc(1)NeuAc(3) |
| HexNAc(5)Hex(3) | HexNAc(6)Hex(6)Fuc(1) |  | HexNAc(6)Hex(7)Fuc(1)NeuAc(1) |
| HexNAc(5)Hex(4) | HexNAc(6)Hex(6)Fuc(1)Neu5Ac(1) |  | HexNAc(6)Hex(7)Fuc(1)NeuAc(2) |
| HexNAc(5)Hex(5) | HexNAc(6)Hex(7)Fuc(1) |  | HexNAc(6)Hex(7)Fuc(1)NeuAc(3) |
| HexNAc(5)Hex(6) | HexNAc(6)Hex(7)Fuc(1)Neu5Ac(1) |  | HexNAc(6)Hex(7)Fuc(1)NeuAc(4) |
| HexNAc(5)Hex(8) | HexNAc(7)Hex(3)Fuc(1) |  | HexNAc(7)Hex(7)Fuc(1)NeuAc(2) |
| HexNAc(6)Hex(3) |  |  |  |
| HexNAc(6)Hex(4) |  |  |  |
| HexNAc(6)Hex(5) |  |  |  |
| HexNAc(6)Hex(6) |  |  |  |
| HexNAc(6)Hex(7) |  |  |  |
| HexNAc(7)Hex(6) |  |  |  |

**Table S6: Lectin family (Pfam Label) details of the promising lectins identified in this study.** The panel of 36 lectins that bound with high intensities to the recombinant SARS-CoV-2 Spike glycoprotein and/or to the SARS-CoV-2 virions were classified as per the Lectin Frontier DataBase (LfDB) ^7,8^.

| **Pfam Label** | **Count** |
| --- | --- |
| Fungal lectin | 1 |
| FB lectin | 1 |
| B lectin | 5 |
| Agglutinin | 1 |
| Jacalin | 4 |
| Gal bind lectin | 2 |
| PA-IIL | 2 |
| Lectin legb | 10 |
| Ricin B lectin | 2 |
| Chitin bind 1 | 1 |
| Unclassified | 7 |

**References**

1 Cheng, K., Zhou, Y. & Neelamegham, S. DrawGlycan-SNFG: a robust tool to render glycans and glycopeptides with fragmentation information. *Glycobiology* **27**, 200-205, doi:10.1093/glycob/cww115 (2016).

2 Wang, L., Nwosu, C., Gao, Y. & Zhu, M. M. Signature Ions Triggered Electron-Transfer/Higher-Energy Collisional Dissociation (EThcD) for Specific and Confident Glycation Site Mapping in Therapeutic Proteins. *J Am Soc Mass Spectrom* **31**, 473-478, doi:10.1021/jasms.9b00101 (2020).

3 Shajahan, A., Supekar, N. T., Gleinich, A. S. & Azadi, P. Deducing the N- and O-glycosylation profile of the spike protein of novel coronavirus SARS-CoV-2. *Glycobiology* **30**, 981-988, doi:10.1093/glycob/cwaa042 (2020).

4 Burke, M. C. *et al.* Determining Site-Specific Glycan Profiles of Recombinant SARS-CoV-2 Spike Proteins from Multiple Sources. *J Proteome Res* **22**, 3225-3241, doi:10.1021/acs.jproteome.3c00271 (2023).

5 Watanabe, Y., Allen, J. D., Wrapp, D., McLellan, J. S. & Crispin, M. Site-specific glycan analysis of the SARS-CoV-2 spike. *Science*, doi:10.1126/science.abb9983 (2020).

6 Chang, D., Klein, J. A., Nalehua, M. R., Hackett, W. E. & Zaia, J. Data-independent acquisition mass spectrometry for site-specific glycoproteomics characterization of SARS-CoV-2 spike protein. *Anal Bioanal Chem* **413**, 7305-7318, doi:10.1007/s00216-021-03643-7 (2021).

7 Hosoda, M. & Angata, K. Databases of lectins (LfDB, LM-GlycomeAtlas, GlyCosmos Lectins-MCAW-DB). . *Glycoforum* **22**, A10 (2019).

8 Yamada, I. *et al.* The GlyCosmos Portal: a unified and comprehensive web resource for the glycosciences. *Nat Methods* **17**, 649-650, doi:10.1038/s41592-020-0879-8 (2020).
